# Interpretable graph-based models on multimodal biomedical data integration: a technical review and benchmarking

**DOI:** 10.1038/s41467-026-74126-5

**Published:** 2026-06-16

**Authors:** Alireza Sadeghi, Farshid Hajati, Ahmadreza Argha, Nigel H. Lovell, Min Yang, Hamid Alinejad-Rokny

**Affiliations:** 1 Holcombe Department of Electrical and Computer Engineering, Clemson University, Clemson, SC USA; 2 School of Science and Technology, Faculty of Science, Agriculture, Business and Law, University of New England, Armidale, NSW Australia; 3 School of Biomedical Engineering, UNSW Sydney, Sydney, NSW Australia; 4 Tyree Foundation Institute of Health Engineering (IHealthE), UNSW Sydney, Sydney, NSW Australia; 5 Shenzhen Institute of Advanced Technology, Chinese Academy of Sciences, Shenzhen, China; 6 UNSW BioMedical Machine Learning Lab (BML), School of Biomedical Engineering, UNSW Sydney, Sydney, NSW Australia

**Keywords:** Data integration, Machine learning, Alzheimer's disease

## Abstract

Integrating diverse biomedical modalities is essential for robust healthcare insights, and graph-based models are increasingly used to capture complex relational structures. Yet, their clinical translation hinges on interpretability. This review surveys interpretable graph-based models applied to multimodal biomedical data, highlighting dominant trends in disease classification, static graph construction, and post-hoc explainability. We categorize explainable artificial intelligence (XAI) techniques, benchmark SHAP, saliency, sensitivity, and graph masking on Alzheimer’s disease data, and reveal complementary strengths. A development flowchart and future directions, such as dynamic graphs, knowledge integration, and LLM-based explainability, position this work as a key reference for trustworthy biomedical AI.

## Abstract (web summary)

The integration of different biomedical modalities in clinical settings is challenging because of limited transparency and explainability. In this review the authors survey and benchmark interpretable graph-based models applied to multimodal biomedical data.

## Introduction

Deep learning (DL) has revolutionized biomedical research through its ability to process complex data and extract meaningful patterns^1^. In healthcare, DL models have improved diagnostics^2^, treatment planning^3^, and drug discovery^4^, while also enabling personalized medicine through predictive risk modeling^5^. However, conventional DL approaches, such as convolutional neural networks (CNNs) and fully connected neural networks, are often limited to homogeneous data and struggle to integrate diverse modalities, like imaging, clinical text, and genomics, critical for comprehensive clinical insights^6–8^.

Graph-based models offer a promising alternative by naturally encoding complex, non-Euclidean relationships via nodes and edges. Beyond applications to single-modality data, such as medical imaging^9–12^ or omics^13–15^, they are particularly well-suited for integrating multimodal biomedical data, capturing both intra- and inter-modality interactions.

Despite these advantages, the adoption of such models in clinical settings is hindered by their limited interpretability^16^. Their “black-box” nature raises concerns around transparency, accountability, and regulatory acceptance^17,18^, highlighting the urgent need for explainable approaches that support clinical trust and informed decision-making.

This review aims to address that need by presenting a comprehensive technical survey of interpretable graph-based models in multimodal biomedical data analysis. Our contributions include: (i) a systematic review of studies incorporating graph-based models and interpretability techniques; (ii) a categorization and benchmarking of explainable AI (XAI) methods; (iii) a step-by-step development guide for building interpretable graph-based models; and (iv) a detailed outlook on future research directions in this rapidly evolving field.

## Survey and benchmarking of XAI strategies

Graph-based models have become integral in analyzing multimodal biomedical data for various regression and classification tasks related to cancer analysis, disease diagnosis, and biomolecular interactions. Table 1 provides an overview of these models, including deep neural networks (DNNs), graph neural networks (GNNs), graph isomorphism networks (GINs)^19^, graph convolutional networks (GCNs)^20^, graph attention networks (GATs)^21^, and graph transformer networks (GTNs)^22^, along with their key characteristics.

**Table 1:** The summary of various graph-based models employed in the surveyed studies, alongside their respective formulas

Model	Explanation	Formula	Denotation
DNN	In graph-related studies, a feedforward framework is built using the nodes and edges of the graph as input.	${z}_{n}=\,{\phi }_{n}({W}_{n}{X}_{n}+{b}_{n})$	${z}_{n}$: the output of the neuron $n$. ${\phi }_{n}$: the activation function applied to the neuron $n$. ${X}_{n}$: the input to the neuron $n$. ${W}_{n}$ and ${b}_{n}$: the weights and bias inputted to the neuron $n$.
GNN	A framework for updating each node’s features by aggregating the features of its neighboring nodes	${{h}_{i}}^{l+1}={{COMB}}^{l}({{h}_{i}}^{l},{{AGG}}^{l}\left(\left\{{{h}_{j}}^{l}:\,j\in {{{{\mathscr{N}}}}}_{i}\right\}\right))$	${{h}_{i}}^{l+1}$ and ${{h}_{i}}^{l}$: node $i$’s feature vector in layer $l+1$ and $l$, respectively. ${{COMB}}^{l}$: the combination function in layer $l$. ${{AGG}}^{l}$: the aggregation function in layer $l$. ${{{{\mathscr{N}}}}}_{i}$: node $i$’th neighborhood.
GIN^19^	An architecture with adjustments to the GNN, to make it more powerful	${{h}_{i}}^{l+1}$ = ${{MLP}}^{l}(\left(1+{\epsilon }^{l}\right).{{h}_{i}}^{l}+{\sum }_{{j}\in {{{{\mathscr{N}}}}}_{i}}\,{{h}_{j}}^{l})$	${{h}_{i}}^{l+1}$ and ${{h}_{i}}^{l}$: node $i$’s feature vector in layer $l+1$ and $l$, respectively. ${{MLP}}^{l}$: a multi-layer perceptron in layer $l$. $\epsilon$: a learnable parameter or a fixed scalar. ${{{{\mathscr{N}}}}}_{i}$: node $i$’th neighborhood.
GCN^20^	A framework for updating the features of graph nodes through convolutional filtering applied to the input graph signals	${H}^{l+1}={\phi }^{l}(A{H}^{l}{\theta }^{l})$	${H}^{l+1}$ and ${H}^{l}$: nodes’ feature vector in layer $l+1$ and $l$, respectively. ${\phi }^{l}$: non-linear activation function of layer $l$. $A$: the adjacency matrix. ${\theta }^{l}$: layer $l$’s convolutional filter’s weight matrix.
GAT^21^	An attention-based architecture for computing the hidden representation of each node by considering the importance of features from other nodes	${\alpha }_{{ij}}={{softmax}}_{j}(a\left(W{h}_{i}\,,{W}{h}_{j}\right))$	${\alpha }_{{ij}}$: attention coefficient of node $j$’s features to node $i$. $a$: the shared attention mechanism (single-layer feedforward NN). ${h}_{i}$: node $i$’s feature vector. $W$: weight matrix of the shared linear transformation
GTN^22^	A framework similar to natural language processing transformers but made for graph inputs	$${{h}_{i}}^{l+1}={O}_{h}^{l}(\begin{array}{c}H\\ \left|\,\right|\\ k=1\end{array}{\sum }_{{j}\in {{{{\mathscr{N}}}}}_{i}}({w}_{i,j}^{k,l}\,{V}^{k,l}\,{{h}_{j}}^{l}))$$	${{h}_{i}}^{l+1}$ and ${{h}_{i}}^{l}$: node $i$’s feature vector in layer $l+1$ and $l$, respectively. ${O}_{h}^{l}$: an additional linear transformation in layer $l$. ||: concatenation operation. $k$: the attention head (from 1 to $H$ heads). ${w}_{i,j}^{k,l}$: the weight of the $k$’th attention head calculated by features from node $i$ and $j$ in layer $l$. ${V}^{k,l}$: the value vector of node $i$ in the $k$’th attention head in layer $l$. ${{{{\mathscr{N}}}}}_{i}$: node $i$’th neighborhood

We also provided a detailed summary of all reviewed papers in Table S1, including the modalities used, graph construction strategies, interpretability techniques applied, and the primary objectives of each study. Additionally, we categories graph-based modeling approaches and the studies in which they were implemented in Table S2.

**Fig. 1:**
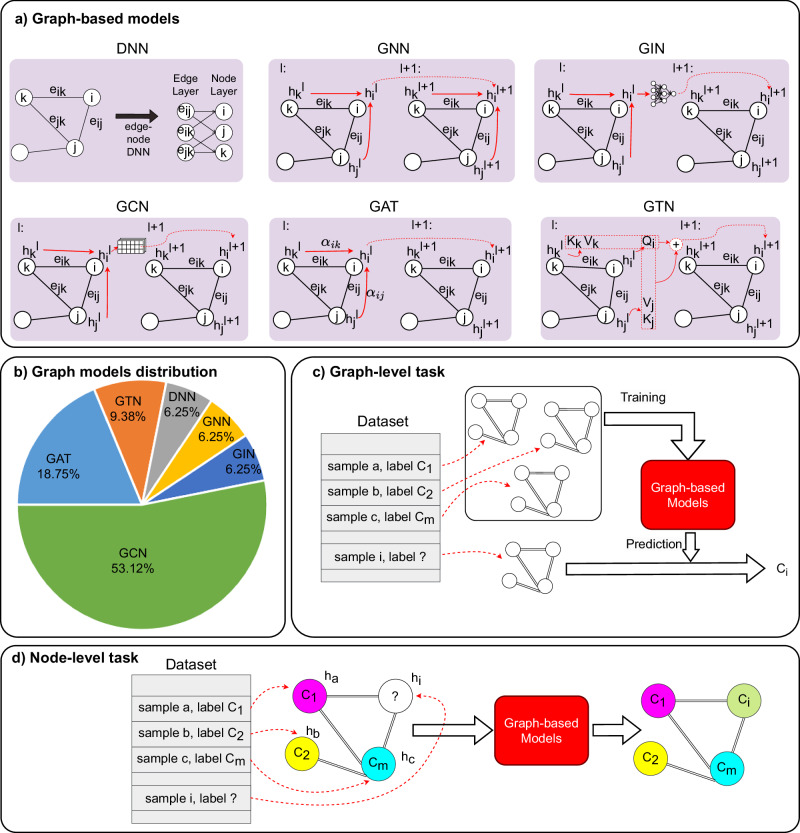
An overview of graph-based models. **a** Architecture showcasing various graph-based models utilized in studies on multimodal data. **b** Distribution of different graph-based models in the reviewed studies (*n* = 31). **c** The process of implementing graph-based models for graph-level analysis. **d** The process of implementing graph-based models for node-level analysis. DNN deep neural network, GNN graph neural network, GIN graph isomorphism network, GCN graph convolutional network, GAT graph attention network, GTN graph transformer network.

Fig. 1a illustrates the architectural schematic of these models, while Fig. 1b displays their distribution across surveyed studies. These models are designed primarily to accomplish two main objectives: performing graph-level tasks or node-level tasks (refer to Fig. 1c, d). In graph-level tasks, each sample in the raw dataset is represented as a graph. Labeled graphs, derived from labeled samples, are used to train the graph-based model. Subsequently, this trained model is employed to make predictions for unlabeled graphs (samples). On the other hand, in node-level tasks, each sample is represented as a node within the constructed graph. The objective is to generate predictions for unlabeled nodes by leveraging information from the labeled nodes in the graph. This approach focuses on predicting individual nodes rather than the entire graph structure.

### Graph construction in the reviewed studies

Graph-based models rely on structured input where a graph ${{{\mathscr{G}}}}={{{\mathscr{(}}}}{{{\mathscr{V}}}},{{{\mathscr{E}}}}{{{\mathscr{)}}}}$, consists of nodes ${{{\mathscr{V}}}}$ and edges ${{{\mathscr{E}}}}$, with node features ${{{\mathscr{F}}}}\left({{{\mathscr{V}}}}\right)$, derived from input modalities. During training, nodes update by aggregating information from neighbors, enabling predictions based on local interactions. Node definitions vary by study objective and modality. Brain imaging studies define brain regions as nodes^23–33^, while genomic and cancer studies use gene or proteins level^29,34–40^, and patient-level prediction tasks (e.g., Alzheimer’s or COVID-19) represent each patient as a node ^41–49^.

Most studies adopt static graph construction, often based on prior biological knowledge (e.g., protein–protein, gene–gene interactions)^34–37,40,50^ or similarity functions like Pearson correlation^23,24,26,29,32,43,51^, Euclidean distance^27,31,44,47,52^, and cosine similarity^32,42,48^. Edges are either thresholded or selected via K-nearest neighbors. Some task-specific strategies also exist, such as Kan et al.’s^30^ dMRI-based connectivity.

**Fig. 2:**
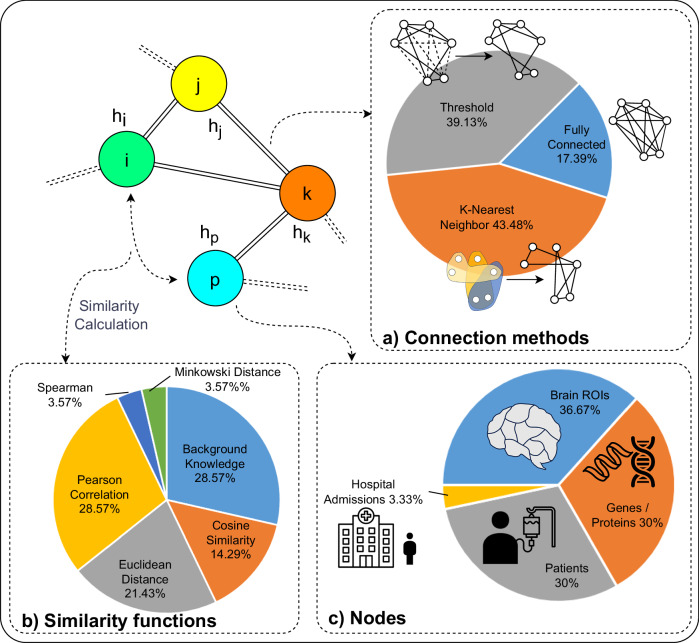
Overview of various techniques employed in different studies to construct graphs for their research. **a** Distribution of various connection methodologies in graph construction utilized by surveyed studies. **b** Distribution of various similarity functions used to draw edges between nodes in the constructed graphs. **c** Distribution of different entities implemented as nodes in the constructed graphs.

A few recent studies explore dynamic graph construction, adapting edge definitions during training to better capture evolving relationships. These dynamic approaches aim to improve performance by overcoming limitations of fixed graph structures.^46,49^. Figure 2 presents an overview of the methods employed by different studies to construct graphs in their research, along with distributions of the techniques utilized.

### Interpretation in multimodal biomedical data integration

DL models, including graph-based ones, are often viewed as black-box systems, which poses a significant challenge when deploying them in trust-sensitive fields like healthcare. To tackle this challenge, researchers in the surveyed studies have explored various approaches to make these models explainable. We examined all the methods implemented in existing studies and categorized them into the following four categories: category I: modality or feature elimination; category II: model-agnostic XAI; category III: graph-based XAI; category IV: inherent interpretability. Table S3 provides an overview of studies that employed one of the aforementioned categories to enhance the model interpretability.

In the following subsections, we explore each category in detail, discussing their specific use cases. For the fourth category, which focuses on developing inherently interpretable graph-based models, we provide a step-by-step guide to assist researchers in creating transparent graph-based models in their studies. Finally, we conclude this section with a benchmark analysis, where we use methods from each category on a specific graph-based medical study to practically evaluate the strengths and weaknesses of each interpretability category.

It is worth noting that beyond the techniques employed in the reviewed studies, there are additional techniques that may fall under the categories of model-agnostic XAI^53,54^, graph-based XAI^55–57^, and inherent interpretability^58–60^ for enhancing the explainability of graph-based models. However, since these techniques were not utilized in multimodal biomedical data integration, they are not included in the following subsections.

#### Category I: modality or feature elimination

**Fig. 3:**
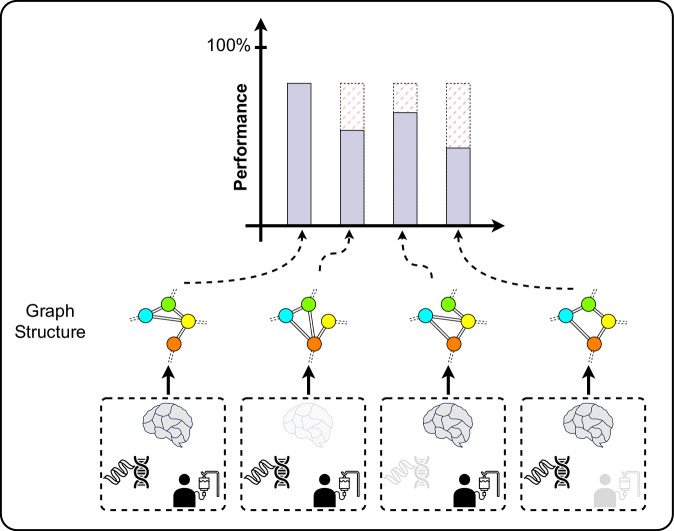
Implementation of the modality elimination technique to assess the importance of each modality in a graph-based model. In this technique, one modality is removed at each step, and the model is retrained using the remaining modalities. This process allows for the calculation of the model’s performance without the influence of the eliminated modalities. By comparing the decline in performance for each modality elimination, insights into the importance of each modality are obtained. Note that eliminating a modality may alter the structure of the graph-based model.

In the context of graph-based models applied to multimodal medical data, a primary focus for researchers is determining the significance of each modality. They seek to understand whether the utilization of multiple modalities enhances their model’s performance compared to scenarios involving a single modality, and if so, to what extent. A common technique employed by most of the reviewed studies involves removing a specific modality and evaluating the model’s performance without the features from that specific modality (Fig. 3). The degree of performance decline indicates the importance of that modality to the model’s overall performance. For instance, Yang et al.^23^ substituted the features from one modality with dummy variables and evaluated the model’s performance, while other reviewed studies opted to eliminate a certain modality, retrain the model, and recalculate its performance to demonstrate the significance of that particular modality.

Although modality or feature elimination is not a classical XAI method, it is sometimes considered under the umbrella of interpretability techniques because it provides empirical evidence of input importance by assessing performance changes upon input removal. Nevertheless, it differs from post-hoc methods that analyze internal model behavior without modifying the input space. However, modality elimination provides only a basic insight into the importance of each modality. For a comprehensive analysis, it is crucial to evaluate the model across all possible combinations of modalities to demonstrate the significance of that particular modality. Moreover, this method serves as an initial step in several model-agnostic XAI techniques like SHAP^61^. Despite some similarities, there are key differences that set them apart. In modality elimination, one modality is removed, and the model is retrained from scratch on the remaining modalities. This retraining process, which focuses exclusively on the remaining features, is not employed in SHAP and similar methods. Therefore, modality elimination can be considered as a distinct category.

Modality elimination can introduce a limitation by potentially altering the graph structure. This alteration is particularly significant when the graph structure relies on the similarity between node feature vectors. Such changes can skew the analysis of modality importance, leading to unfair conclusions. To delve into it further, let us consider a feature vector ${{{\mathscr{F}}}}\left({{{{\mathscr{V}}}}}^{n}\right)=\left\{{f}_{1}^{n},\,{f}_{2}^{n},\,\ldots,\,{f}_{m}^{n}\right\}$, where ${{{\mathscr{F}}}}\left({{{{\mathscr{V}}}}}^{n}\right)$ represents the feature vector of the $n$-th node and ${f}_{i}^{n}$ represents features derived from the $i$-th modality. To gauge the effect of eliminating the $i$-th modality, we create a new feature vector ${{{\mathscr{F}}}}\left({{{{\mathscr{V}}}}}_{-i}^{n}\,\right)=\left\{{f}_{1}^{n},\,{f}_{2}^{n},\,\ldots,\,{f}_{i-1}^{n},{{f}}_{i+1}^{n},\ldots,\,{f}_{m}^{n}\right\}$ which includes information from all modalities except the $i$-th one. After creating this new feature vector for all nodes, the similarity between nodes will change. This means that nodes $n$ and $k$ that were initially similar may no longer be similar, and vice versa:

1 $$\left\{\begin{array}{l}{Sim}\left({{{\mathscr{F}}}}\left({{{{\mathscr{V}}}}}^{n}\right),{{{\mathscr{F}}}}\left({{{{\mathscr{V}}}}}^{k}\right)\right)=\alpha < \tau \Rightarrow {{{{\mathscr{V}}}}}^{n},{{{{\mathscr{V}}}}}^{k}{neighbors}\\ {Sim}\left({{{\mathscr{F}}}}\left({{{{\mathscr{V}}}}}_{-i}^{n}\right),{{{\mathscr{F}}}}\left({{{{\mathscr{V}}}}}_{-i}^{k}\right)\right)=\alpha > \tau \Rightarrow {{{{\mathscr{V}}}}}^{n},{{{{\mathscr{V}}}}}^{k}{not}\,{neighbors}\end{array}\right.$$

Here, ${{{\mathrm{Sim}}}}\left(\cdot \right)$ represents the similarity function between two vectors, and $\tau$ represents the threshold below which nodes with similarity values are considered neighbors. Researchers face two choices in this context: (i) they can reconstruct the graph structure based on the new similarities, or (ii) they can retain the original graph structure and continue with the analysis. In the first scenario, where the graph structure is reconstructed, comparing the performance of the graph-based model on this new graph with the original one would not provide a fair assessment of the eliminated modality’s impact. However, in the second scenario, maintaining the structure of the initial graph poses a risk of connecting dissimilar nodes. This could confuse the model by incorporating information from irrelevant neighbors, potentially leading the model away from its optimal performance. Therefore, removing modalities in graph-based studies, particularly when edges are determined by node features, is not a fair method for assessing the impact of each modality.

#### Category II: model-agnostic XAI

**Fig. 4:**
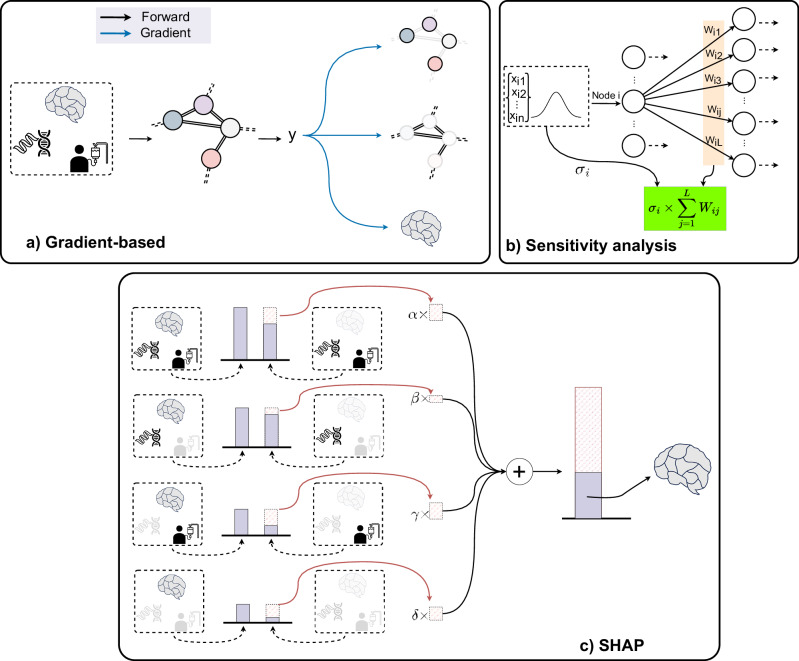
An overview of various model-agnostic XAI techniques used in graph-based studies to gain insights from the model. **a** Gradient-based method: this technique is used to assess the importance of different components in a graph-based model. It involves calculating the gradient of the model’s predicted value with respect to various elements, such as graph nodes, edges, input features, or modalities. By analyzing these gradients, researchers can identify which components most significantly influence the model’s predictions. **b** Sensitivity analysis: this is a common method for determining the importance of features in a neural network. It involves calculating the standard deviation of a specific feature across all samples and multiplying it by the sum of the weights associated with that feature in the trained network. This product reflects the feature’s overall importance to the model’s performance. **c** SHAP: SHAP is a widely used method for enhancing model interpretability. It calculates the model’s performance with and without specific modalities (e.g., brain ROIs on the left side). The difference in performance when a modality is included versus when it is excluded is computed across all possible modality combinations. These differences are then combined linearly to quantify the contribution of each modality to the model’s overall performance.

In addition to evaluating the importance of each modality, several studies have focused on determining the significance of various components of their constructed graph. This includes assessing the importance of an individual node, the edges between nodes, and features derived from different modalities for each node. To achieve this, researchers have employed several post-hoc XAI techniques originally developed for general machine learning, rather than specifically for graph-based models. For example, the saliency map technique^62,63^, which relies on gradient calculation, was used by several studies^26,28,29,51^ to assess the importance of each node’s feature. Bi et al.^29^ also used this technique to determine the importance of graph nodes, where each node represents a specific brain ROI. Another gradient-based XAI technique, which integrated gradient analysis^64^, was used by Huo et al.^39^ to unveil the importance of each genomic feature employed in their study. Additionally, Qu et al. used Grad-RAM^24^, another gradient-based technique, to identify the importance of each node in the graph, where each node also represents a specific brain ROI. Qu et al.^24,33^ further provided edge-level interpretability using an edge-masking technique, which involved systematically removing one edge at a time from all patient graphs and retraining the model to observe the resulting performance changes, thereby determining the significance of each edge. Zhang et al.^35^ and Pfiefer et al.^37^ used SHAP^61^, a technique based on Shapley values, to illustrate the importance of each feature and node, respectively. Sensitivity analysis^65^, a common technique to determine feature importance in neural networks, by multiplying network weights and feature standard deviation, was used by Li et al.^44^ to identify feature importance in their study. Figure 4 provides detailed visualizations of each of these techniques. Table S4 summarizes the various model-agnostic XAI methods along with their corresponding formulae employed in different studies.

#### Category III: graph-based XAI

While post-hoc model-agnostic XAI techniques can offer valuable insights into the importance of various components in graph-based studies, it is important to recognize their limitations. Gradient-based XAI techniques, for example, have been shown to be misleading in certain scenarios^66^ and can suffer from issues like gradient saturation^64,67^. Additionally, these techniques are not specifically designed for graphs where inputs are often discrete, which further limits their effectiveness when applied to graph-based models^68^. To overcome these challenges, researchers have employed XAI techniques specifically tailored for graph-based models.

**Fig. 5:**
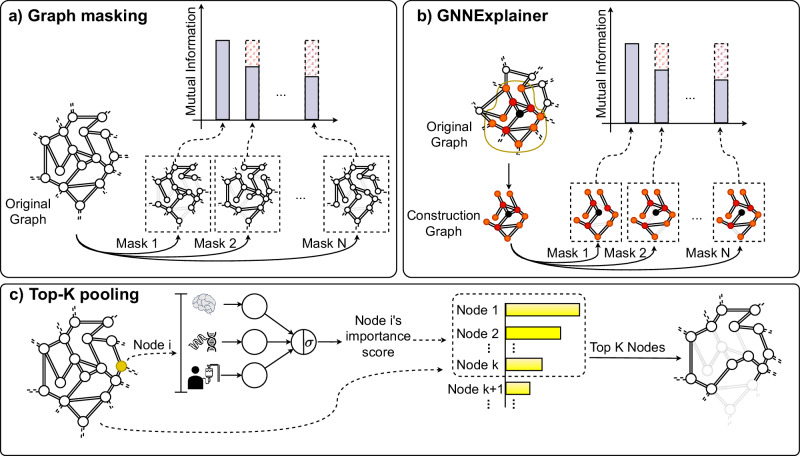
An overview of graph-based XAI techniques used to gain insights from graph-based models. **a** Graph masking: this technique involves applying a mask (either an edge mask or a feature mask) to the original graph structure. The mutual information between the masked graph and the true label distribution is then calculated. The masked graph with the highest mutual information value is identified as the most important or informative graph. **b** GNNExplainer: similar to the graph masking method, GNNExplainer applies masks to the graph to uncover important components, such as nodes, edges, or features, that significantly influence the model’s predictions. **c** Top-k pooling: in this method, each node’s features are passed through a single-layer MLP to calculate the importance of each node. The nodes are then ranked based on their importance scores, and the top-k nodes are selected to construct the final graph. XAI eXplainable artificial intelligence, GNN graph neural network, MLP multi-layer perceptron.

Zhou et al.^27^ employed a general graph masking technique to identify the most significant subgraph and subset of features within their model. They specifically developed optimal masks for both the graph structure and the feature vector, allowing them to distinguish the most important subgraph and feature subset. Additionally, GNNExplainer^68^, another masking-based technique, was employed by Pfeifer et al.^36^, Tang et al.^52^, and Patel et al.^32^ to pinpoint the most crucial nodes and features within a graph during the prediction process for a specific sample. The primary difference between the general graph masking technique and GNNExplainer lies in their scripts. While GNNExplainer focuses on the construction graph of a particular node, essentially a subset of the original graph that directly influences the output for that node, the general graph masking technique considers the entire original graph. GNNExplainer offers computational advantages, especially in practical applications involving large graphs with potentially millions of nodes. Another technique used to determine the importance of each node in an input graph is the top-k pooling technique^69^. In this method, the importance score of graph nodes is computed using multi-layer perceptron (MLP) models. This approach, which offers node-level interpretability, was applied by Sebenius et al.^25^ to brain ROIs to identify the most important ROIs for diagnosing patients with schizophrenia. The details of the graph-based XAI techniques are also demonstrated in Fig. 5, while Table S5 summarizes different graph-based XAI techniques along with their corresponding formulae employed in the surveyed studies.

#### Category IV: inherent interpretability

While post-hoc XAI techniques are valuable for gaining insights into the components of complex models, interpretable models offer inherent transparency by allowing tracking of the data-processing steps. This transparency enables understanding of how different components collaborate to generate predictions, clearly showing how features, nodes, and edges contribute to the model’s outputs. In the realm of multimodal medical data integration using graph-based models, several studies have developed inherent interpretable models. The elements used in these studies can be classified into the following three categories.

*Attention mechanism*: several studies integrated attention mechanisms, which assign higher attention weights to more important components. In the study conducted by Yang et al.^23^, graph inputs are first processed through a multi-head attention layer to compute attention weights for each node. By employing gradient sensitivity analysis to these weights, the study reveals specific features and their interactions pertinent to the classification process. In another study, Ouyang et al.^49^ introduced an attention layer within their model to elucidate the significance of each modality. They also incorporated inner product regularization on the feature indicator matrix to emphasize the more critical features within each modality. Similarly, Huo et al.^39^ also employed attention mechanisms to identify significant clinical and pathological features within the corresponding graphs in their study. In another work, Ma et al.^40^ utilized the attention mechanism in their model to determine the importance of genes within specific cells. Kazi et al.^46^ developed IAM, an attention-based component in their model designed to reveal the importance of each input feature. Additionally, the authors introduced a novel loss function tailored specifically for this component, enhancing the IAM’s ability to detect more important features. Lastly, in the model proposed by Bintsi et al.^45^, features are initially processed through an attention layer. Subsequently, the weighted features, constructed by multiplying feature values, and the weighted features—created by multiplying feature values by their corresponding attention weights—serve as the feature vector for each node (patient). Once the model is fully trained, the attention weights reveal the importance of each feature, identifying the most crucial ones.

*Graph attention*: beyond the general attention layers used in the above studies, some researchers incorporated graph-specific attention mechanisms to enhance the interpretability of their model. In the study by Keicher et al.^41^, they used a GAT^21^, where the attention weights shed light on how neighboring nodes (patients) influence the classification of a specific node (patient). This approach is particularly valuable in pandemic scenarios, such as the COVID-19 study they conducted, offering valuable insights into population interactions and potential outcomes for individual patients. Similarly, Safai et al.^28^ also employed GAT to understand the significance of nodes (brain ROIs) in the model’s prediction, aiming to elucidate the interactions between various brain regions. Additionally, Li et al.^50^ used GAT to identify the importance of different nodes (drug-protein pairs) in their study. Xiao et al.^48^ leveraged GAT in their proposed model to determine each node’s (patient) contribution to the prediction of outcomes for other patients. Additionally, Kaczmarek et al.^22^ used the graph transformer, another graph-specific attention mechanism, to analyze the significance of interactions between nodes (specifically mRNA–miRNA interactions) in detecting various types of cancers. They further calculated the average edge weights associated with each node to identify key nodes (mRNA or miRNA) relevant to specific cancer classification tasks.

*Other methods*: some researchers have developed interpretable graph models without relying on attention. For example, Kan et al.^30^ used network weights to evaluate node and feature importance, while Bi et al.^47^ assessed filter weights in graph convolution layers. Pfeifer et al.^37^ integrated decision trees, leveraging their inherent interpretability to highlight modality and feature contributions.

Model interpretability strategies often depend on the study’s objective.

### Benchmarking existing strategies

We conducted a benchmarking analysis to evaluate the performance and practicality of the previously described XAI techniques when applied to a graph-based model. Specifically, we focused on MOGONet, a graph-based model developed by Wang et al.^42^ for biomedical classification tasks across various diseases. In our study, we replicated the MOGONet architecture using the ROSMAP dataset^70^, which is curated for Alzheimer’s disease (AD) classification. The dataset comprises features from three distinct modalities, including mRNA expression data, DNA methylation data, and microRNA expression data, each processed through its respective graph-based model within the MOGONet framework. Final classification outcomes were obtained by integrating the predictions generated by these individual models.

To assess feature importance, the original study in MOGONet employed a modality elimination technique (category I), wherein the performance impact of excluding specific features was measured. Building on this approach, we extended the analysis by implementing additional XAI techniques from categories II and III to gain deeper insights into the relative significance of the dataset’s features. Category IV methods were excluded from this analysis, as these inherently interpretable models are not model-agnostic and thus cannot be retrofitted onto pre-existing architectures like MOGONet. For this benchmarking analysis, we selected gradient saliency Map, SHAP, and sensitivity analysis from category II. From category III, we incorporated graph masking, a generalization of GNNExplainer, into the analysis due to its broader applicability in identifying important features and subgraphs. However, the top-k pooling method, which is specifically designed to assess node importance, was excluded as it is not directly applicable to feature-focused analyses.

After training, we used the trained model’s parameters and the feature distributions from the test set to calculate the importance of each feature using sensitivity analysis. This technique was relatively fast because it didn’t require backpropagation or additional model training. To compute feature importance using gradient saliency maps, we calculated the gradient of the model’s output for each test sample with respect to each feature. The feature importance was revealed by averaging these gradients across all test samples. For the graph masking technique, we aimed to find a mask that, when applied to the graph, maximized mutual information with the non-masked graph. We started with a randomly initialized mask and optimized it over 100 epochs using the binary cross-entropy cost function. This method was more time-consuming than the previous two, as we needed to find the optimal mask for each test sample individually. Finally, we used Kernel SHAP to compute feature importance. We generated 100 weighted neighbors for each test sample, with the weights calculated using the equations proposed by Lundberg et al.^61^. By training a logistic regression model on these generated samples, we determined the feature importance for each sample. For global interpretability, we averaged these importance values across all patients.

**Fig. 6:**
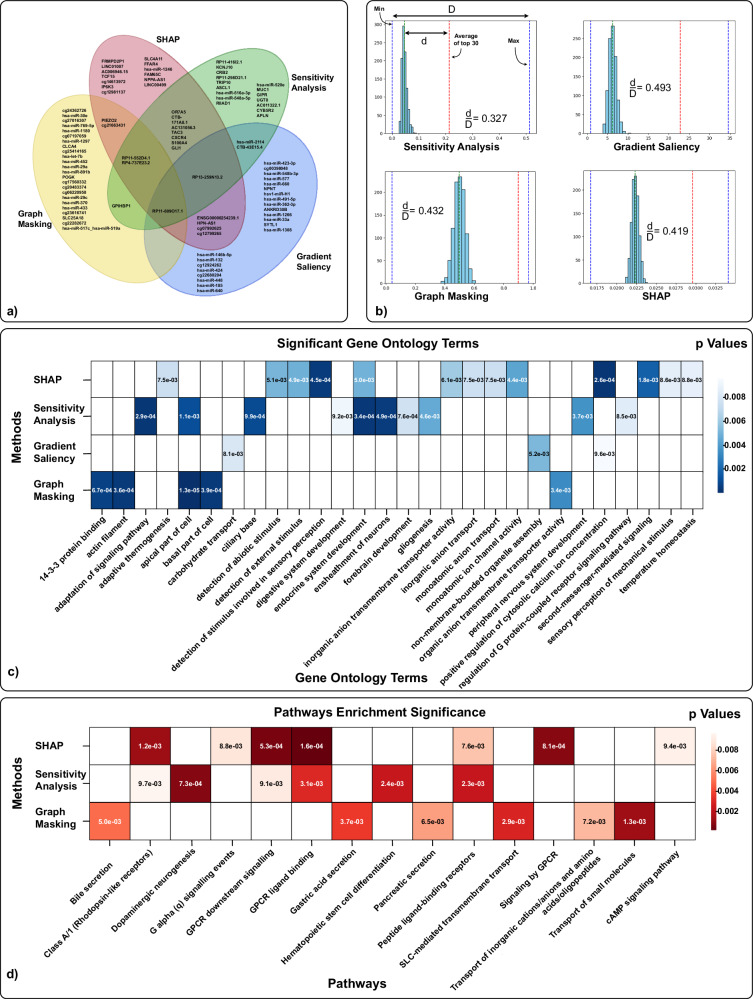
Benchmarking analysis. **a** Top 30 features captured by different XAI techniques from categories II and III are considered the most important features for Alzheimer disease classification. As shown in the figure, SHAP and gradient saliency were roughly able to detect a combination of the most important genes found by other techniques, as the most important genes for Alzheimer disease classification. **b** A permutation analysis was performed on the important features identified by each technique. Specifically, 30 features were randomly chosen from the entire feature set, and their average importance scores were calculated. This random selection process was repeated 1000 times, creating a distribution of average importance scores, which was visualized in a histogram. The average importance score of the top 30 ranked features (shown by a red dotted line) was then compared to the mean of the permutation-based score distribution to assess the significance of these selected features. The results show that the gradient saliency technique outperformed the other techniques. **c** In the GO analysis, we identified several GO terms, retaining only those with a *p* ≤ 0.01. These terms, along with their *p*-values and the XAI techniques that identified them, are shown. **d** Pathway analysis was also conducted, keeping only pathway terms with a *p* ≤ 0.01 and an overlap >1; notably, none of these terms were identified by gradient saliency. The retained pathway terms, the XAI techniques that found them, and their *p*-values are displayed. XAI eXplainable artificial intelligence, SHAP SHapley Additive exPlanations GO gene ontology.

We first compared the top genes identified by each XAI method. As Fig. 6a showed, all four methods—SHAP, sensitivity analysis, gradient saliency, and graph masking—identify partially overlapping yet distinct sets of top genes, reflecting the inherent variability in how each explainability algorithm quantifies feature importance. SHAP and sensitivity analysis share more genes in common (e.g., *CTB-171A8.1*, *RP11-552D4.1*, and *OR7A5*), consistent with both methods having a relatively broad coverage of biologically relevant signals. Gradient saliency highlights genes, such as *NPNT*, *SYTL1*, and *ANKRD30B*, which do not appear prominently in the other lists, suggesting their sensitivity to different aspects of model gradients. Graph masking surfaces genes associated with membrane transport and cell signaling (*SLC25A18*, *GPIHBP1*, *CLCA4*, and *SCD*), reflecting its unique strategy of learning a mask that pinpoints the most relevant subsets of the graph structure.

To assess how each interpretability method’s top 30 genes compared against random chance, we calculated the average importance score of those top 30 genes and then generated a null distribution by repeatedly (1000 times) sampling 30 genes at random (Fig. 6b). In the figure, the ratio indicates how far this observed average lies from the mean of the random distribution. Notably, gradient saliency shows the largest offset, implying that its top-ranked genes are especially unlikely to have been identified by chance, whereas sensitivity analysis yields a smaller offset but still remains distinctly above the random null distribution. Graph masking and SHAP occupy intermediate positions, further underscoring that each algorithm captures important gene sets in different ways.

We then conducted gene ontology (GO) analysis on the top genes identified by each XAI technique and extracted the corresponding GO terms, keeping only those with *p* ≤ 0.01. Figure 6c presents the GO terms alongside the XAI techniques that identified them. As the figure shows, SHAP identified ten out of eighteen Alzheimer-related GO terms (unique GO terms identified by all methods), while sensitivity analysis identified six. In comparison, graph masking and gradient saliency each detected only two Alzheimer-related GO terms. Interestingly, each XAI method highlighted distinct yet sometimes overlapping GO terms relevant to AD. SHAP detected multiple AD-associated processes, including *positive regulation of cytosolic calcium ion concentration*, *endocrine system development*, and *temperature homeostasis*, each tied to neuronal dysfunction and metabolic dysregulation in AD. Sensitivity analysis captured partially overlapping GO terms (e.g., *endocrine system development*) while adding *ensheathment of neurons*, *gliogenesis*, and *forebrain development*, reflecting broader coverage of neurodevelopmental processes. Gradient saliency identified fewer terms, but still pinpointed *positive regulation of cytosolic calcium ion concentration* and *carbohydrate transport*, both linked to neuronal health and metabolic deficits in AD. Graph masking introduced unique findings like 14-3-3 *protein binding*, relevant to tau pathology, and *actin filament*, associated with cytoskeletal disruptions.

We then repeated the same analysis on the 15 unique pathway terms identified by all the methods (we only considered those pathways with *p* ≤ 0.01). Notably, none of these retained pathways were identified by gradient saliency; seven were identified by SHAP, while both sensitivity analysis and graph masking methods identified six pathways (Fig. 6d). SHAP and sensitivity analysis both prominently identified pathways involving G protein-coupled receptor (GPCR) signaling (e.g., GPCR ligand binding, GPCR downstream signalling, class A/1 rhodopsin-like receptors, signaling by GPCR), processes closely linked to synaptic transmission and neurodegeneration in AD. Sensitivity analysis, in particular, highlighted dopaminergic neurogenesis and hematopoietic stem cell differentiation, reflecting broader aspects of neuronal and immune system development relevant to AD. SHAP, meanwhile, singled out the cAMP signaling pathway, another key mediator of neuronal plasticity and survival that has been implicated in AD pathophysiology. In contrast, graph masking emphasized more general transport pathways (e.g., SLC-mediated transmembrane transport, bile secretion, gastric acid secretion) and metabolic processes, which may indirectly relate to AD through systemic metabolic dysregulation but are less directly established in AD literature compared to GPCR signaling or dopaminergic function.

## Discussion, perspective, and future directions

**Fig. 7:**
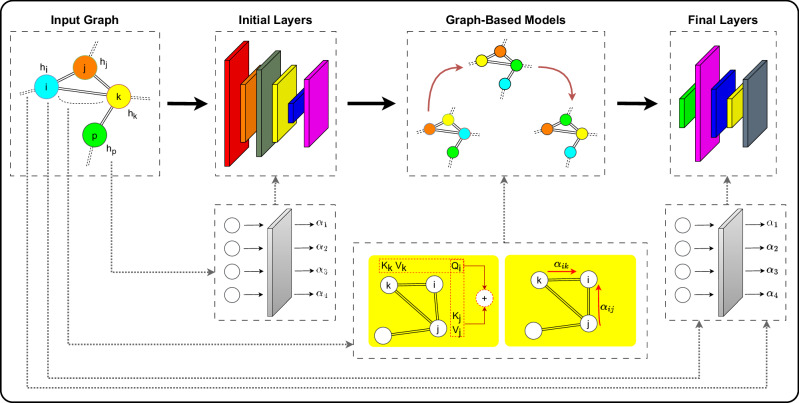
A general guideline for developing an interpretable graph-based platform on multimodal medical data. It is important to note that the purpose of interpretability and the study’s objective significantly influence the type of elements to use and their placement within the model.

In this paper, we examined how graph-based models support multimodal medical data integration and enhance interpretability in tasks, such as disease classification. Through benchmarking various explainability techniques, ranging from post-hoc XAI to inherently interpretable models, we highlighted trade-offs between computational efficiency and explanatory depth. While gradient- and sensitivity-based methods offer quick insights into feature importance, techniques like SHAP and graph masking yield richer interpretations at a higher computational cost. Figure 7 presents a practical framework that we propose to guide researchers in integrating explainability into graph-based biomedical models. For node- and edge-level interpretability, attention mechanisms should be positioned closer to the output layers to highlight the components most influential in the final prediction. Conversely, for feature-level interpretability, attention layers are best applied earlier in the architecture to capture the relevance of input features.

Building on this framework, models, such as GAT and GTN, offer practical implementations that help visualize node interactions and their predictive contributions. However, while inherently interpretable models can enhance transparency, they often require architectural simplifications that may reduce predictive performance. This highlights the inherent trade-off between model accuracy and interpretability. Currently, most studies still depend on general-purpose XAI tools like SHAP and gradient-based saliency methods. Only a limited number of works employ graph-specific interpretability approaches, such as GNNExplainer or construct models with built-in interpretability mechanisms. Researchers are encouraged to adopt a balanced approach, selecting methods aligned with the interpretability needs of their application, the complexity of the data, and the clinical or biological questions at hand.

In addition to architectural challenges, practical deployment must also consider the efficiency and feasibility of interpretability methods. In real-world settings, limited time and computational resources necessitate careful selection of post-hoc XAI techniques. For instance, SHAP, while offering deep interpretability, can be prohibitively slow, taking weeks for high-dimensional data. As a result, inherently interpretable models are gaining traction. However, these often trade accuracy for transparency, requiring researchers to balance performance needs, computational costs, and interpretability demands^71^. Hybrid models, blending attention mechanisms or lightweight post-hoc methods, have shown promise in mitigating this trade-off^72–74^. In clinical applications, where actionable insights are critical, opting for higher interpretability at the expense of slight accuracy loss may be justified^75^. Crucially, interpretability is not just a technical preference; it underpins trust, accountability, and safety in healthcare by clarifying model rationale and supporting clinical decision-making.

Beyond the design of individual models, recent innovations in how biomedical data is structurally represented also shape interpretability and model utility. To extend the utility of these graph models beyond traditional tasks, recent advances have proposed “graph-in-graph” strategies. In this setup, each data sample (e.g., a patient) is both a node in a global graph and a graph itself, capturing detailed molecular or imaging data. This hierarchical representation enables simultaneous patient-level and system-level reasoning, which can uncover patterns otherwise lost in flat graph structures^76^. For example, in cancer studies, each patient node could contain a subgraph of gene interactions or imaging-derived features. This dual-layer architecture allows researchers to identify both local disease markers and broader trends across the population, providing a richer and more nuanced understanding of disease progression and treatment response.

While these architectural innovations expand modeling capability, fundamental technical challenges remain in deep graph learning. Deep graph models often suffer from over-smoothing, where node features become indistinguishable with increased message-passing layers. Rusch et al.^77^ showed that addressing this issue is vital for model performance. Another limitation, over-squashing, arises when information from distant nodes is compressed, impairing long-range dependency learning. Topping et al.^78^ linked this to graph topology and proposed an edge-based combinatorial curvature method as a mitigation strategy.

Despite technical progress, the application of these models remains uneven across disease types and tasks. While interpretable graph-based models have shown promise in cancer analysis^34,36,38,39^, current studies largely focus on a few cancers like breast^35,44^ and kidney^37^, leaving others, such as prostate, skin, and thyroid, underexplored. Similarly, their application to disease diagnosis has been limited to COVID-19 [19^41^, Alzheimer’s^27,29,46,47^, and Parkinson’s^28,43^, highlighting opportunities to extend these methods to diseases like malaria, AIDS, and hepatitis. Most existing models address classification or regression tasks; expanding to image segmentation or object detection could uncover new challenges and validate generalizability. Such diversification requires flexible graph architectures capable of integrating heterogeneous data, e.g., imaging, sequences, and clinical variables, especially in settings where multimodal data availability and quality vary significantly. Table S6 summarizes disease areas explored to date.

One factor that influences both performance and interpretability is how graphs are constructed. Existing studies have constructed graphs using either domain knowledge^34–38^ or similarity functions, such as cosine^48,49^, Euclidean^44,46,47,52^, and Pearson correlation^24,26,29,43^. Future work could leverage multimodal knowledge graphs or hybrid approaches combining knowledge and similarity-based edges. Exploring dynamic graph construction, where edges adapt based on disease context or patient subgroups, could uncover hidden patterns. While the effects of static vs. dynamic construction on explainability^79^ and performance^80^ have been studied individually, their combined impact remains underexplored. Additionally, tracking the evolution of the graph structure during the training process can provide insights into how the model updates over time, potentially guiding improvements in performance. Visualization tools, such as NetworkX with Matplotlib animation (Python), GraphStream (Java), or dg2pix^81^ (a pixel-based method), can support this type of analysis.

While some studies have adopted post-hoc explainability tools specifically designed for graph networks, such as GNNExplainer^68^, the field still underutilizes other advanced graph-specific XAI techniques. Recent developments, including PGExplainer^82^ and CF-GNNExplainer^83^ offer promising alternatives that go beyond simple node- or edge-level attribution by identifying minimal subgraphs or generating counterfactual explanations. These methods can be more adaptable to diverse graph structures and potentially yield deeper, more localized biological insights. Future research should explore these alternatives more systematically, particularly in multimodal biomedical applications, where interpretability at the subgraph or pathway level is critical.

To fully realize the potential of these advanced techniques, rigorous evaluation frameworks must be adopted. Most reviewed studies used explainability methods and qualitatively aligned their results with prior knowledge (e.g., ground truth). However, quantitative evaluation offers a more objective assessment of how well these techniques highlight meaningful model components. When ground truth is available, metrics like accuracy, F1, precision, specificity, and sensitivity can be used^53,68^. In its absence, metrics, such as fidelity, inverse fidelity, and sparsity, are preferred^57^. Fidelity measures the performance drop after removing important components; inverse fidelity evaluates performance using only those components; and sparsity penalizes overly large explanations. Ideally, effective methods achieve high fidelity, high sparsity, and low inverse fidelity (see more in the “Methods section”).

While our reviewed studies employed spatial GNNs, where node representations are iteratively updated via message passing, it is equally important to consider spectral GNNs, which operate in the frequency domain using graph Fourier transforms. These models transform input signals into the spectral domain via the graph Laplacian, apply a learnable filter, and then invert the result back to the spatial domain. The effectiveness of spectral GNNs largely depends on the design of the filter, which modulates how frequency components are emphasized^20,84^. Bo et al.^85^ benchmarked different spectral filters across datasets, showing that while more advanced filters can enhance performance, they also introduce significant computational complexity, often limiting their practical adoption. Nonetheless, as spectral models mature, they may offer complementary strengths to spatial methods, especially in capturing global graph structures.

Complementing spatial GNNs, spectral approaches offer alternative mechanisms for learning and interpreting graph structure. The explainability techniques reviewed earlier are largely tailored to spatial GNNs and are not directly applicable to spectral-based models. However, spectral GNNs offer intrinsic interpretability via their frequency-domain representation: the eigenvectors of the graph Laplacian define frequency components that the model selectively amplifies through learnable filters. Low, mid, and high frequencies, respectively, capture global patterns, balanced structures, and local variations. While this spectral insight offers a unique lens into model behavior, it lacks direct spatial interpretability, crucial for clinical applications. Bridging this gap remains an open research challenge in multimodal biomedical explainability.

Our benchmarking revealed that each XAI method offers distinct advantages in uncovering Alzheimer’s disease (AD)-related biology. SHAP and sensitivity analysis consistently retrieved well-established GO terms and pathways, such as GPCR signaling, neurogenesis, and protein binding, making them particularly valuable for studies prioritizing biological interpretability. In contrast, gradient saliency and graph masking surfaced alternative insights, including 14-3-3 protein binding and metabolic transport, highlighting underexplored aspects of AD. Permutation testing confirmed that all methods outperformed random baselines, with gradient saliency producing uniquely distributed importance scores. Computationally, SHAP and graph masking were more resource-intensive, while gradient saliency and sensitivity analysis ran faster but offered more limited biological overlap. These findings suggest that combining multiple XAI approaches may yield a more comprehensive and nuanced understanding of AD pathophysiology by balancing interpretive depth with efficiency.

However, challenges remain. Many reviewed studies focus narrowly on specific diseases or modalities and rely heavily on qualitative interpretations, limiting generalizability and rigor. In our benchmarking, the absence of human-annotated ground truth for important graph components constrained direct validation. Furthermore, while this review covered a broad range of multimodal studies, excluding single-modality work and computational bioinformatics may have overlooked relevant progress. Future work should strive to bridge these areas for a more unified framework of explainable graph learning.

Emerging XAI methods, such as counterfactual reasoning^86^ and contrastive explanations^87^, offer promising directions by identifying minimal, actionable changes to node features or edges that shift model outputs. These techniques have the potential to enhance model transparency and support precision interventions, an essential step for translating graph-based models into high-stakes clinical settings.

### Future directions: LLM-integrated graph models

Integrating large language models (LLMs) into graph-based biomedical analysis offers a promising step toward more dynamic and interpretable multimodal AI. LLMs excel at extracting structured knowledge from unstructured text, such as clinical notes^88^, scientific literature^89^, and patient records^90^, and can enhance graph construction by identifying relevant entities and relationships for node and edge definition^91^. This enables the development of context-aware, dynamically updated graphs that move beyond static similarity-based connections.

LLMs also show potential in improving interpretability by generating natural language explanations. While current graph-specific XAI methods like GNNExplainer^68^ or graph masking provide numerical or visual outputs, they often lack semantic context. Prompting LLMs to explain model-predicted subgraphs or node clusters could bridge this gap, linking model insights to known pathways, disease mechanisms, or drug–gene interactions. This approach can make graph explanations more intuitive and clinically meaningful, fostering trust and utility among healthcare stakeholders.

Beyond summarizing background knowledge, LLMs can enhance XAI by converting graph-based explanations into human-readable narratives that clarify not just what influenced a prediction, but why. This “explanation chaining” approach, illustrated in other domains by Huang et al.’s chain of explanation (CoE) prompting^92^, could be adapted for biomedical graphs to narrate how a sequence of molecular or clinical interactions leads to a specific diagnosis.

LLMs can also provide meta-interpretations by explaining outputs from other XAI methods. For instance, they can articulate why SHAP scores highlight certain features or why a graph masking method identifies a particular subgraph. Gao et al.‘s DR.KNOWS^93^. exemplifies this by translating medical knowledge graphs into diagnostic narratives. Similarly, He et al.^94^ used LLMs to generate and process textual explanations in graph-based learning, enhancing node representations and transparency. Zhang et al.^95^ further showed how combining Grad-CAM with multimodal LLMs improves both interpretability and performance. Collectively, these approaches signal a future where LLMs serve as bridges between complex graph analytics and clinician-friendly, multimodal interpretability frameworks.

LLMs can generate semantic embeddings from biomedical literature that, when aligned with numeric features like gene expression or imaging metrics, enhance graph-based models by incorporating both statistical and contextual relevance^96^. For example, two genes may be co-expressed and also frequently co-mentioned in Alzheimer’s studies, reinforcing their role in GNN message-passing.

LLM–GNN integration also enables interactive querying, where clinicians can ask questions like “what markers predict advanced Parkinson’s disease?” and receive subgraph-based, literature-backed answers, bridging the gap between expert insight and model output. However, several challenges remain. LLMs are computationally intensive to train or fine-tune^97^, and may generate false or unsupported information (“hallucinations”)^98^, highlighting the need for validation pipelines. Privacy concerns in handling sensitive clinical data necessitate strong anonymization, while biases in training corpora raise fairness issues in clinical applications ^99^.

In conclusion, advancing explainable, multimodal GNN frameworks, particularly through LLM integration, offers transformative potential, but it still requires careful validation, ethical safeguards, and methodological innovation to ensure reliable clinical translation.

## Methods and materials

### Methodology

To gather relevant research papers, we used three primary sources: Google Scholar, Scopus, and PubMed. We conducted research using various combinations of the following keywords related to our study:

- Interpretable, explainable, or transparent graph networks
- Multimodal, integrated, multimodality, cross-modal, multi-source, integration, fusion, or heterogenous
- Medical, biomedical, biological, healthcare, genetic, genomic data, clinical notes, electronic health records, neuroimaging, MRI, fMRI, PET, CT, proteomic, transcriptomic, metabolomic, pathology, radiology, or omics. Summary of the diverse biomedical data types used across the reviewed studies has been provided in Table S7.

An example of the queries used to identify the papers is provided in Table S8. This comprehensive search yielded a total of 3129 papers published between January 1, 2019, and June 30, 2025. Subsequently, we screened these papers by their titles, leading to the inclusion of 401 papers for further examination. The abstracts of these selected papers were reviewed in the next step, resulting in the identification of 138 papers for a more in-depth analysis. Upon analyzing the full-length papers, 107 of them were excluded for various reasons. Some papers were duplicates, others used graph-based models in different contexts unrelated to multimodal data analysis, and some failed to provide a clear explanation of the interpretability techniques employed in their models. This filtering process left us with 31 papers eligible for final review and inclusion in our study. To illustrate our selection process, we have depicted a PRISMA diagram in Fig. S1, outlining the stages of paper selection and exclusion. It is important to note that each step of the methodology described was conducted independently by all authors involved in this study.

### Gene ontology and pathway analyses

We employed over-representation analysis (ORA) via WebGestalt (https://2019.webgestalt.org/)^100^ to investigate gene ontologies and biological pathways associated with protein-coding genes. This approach aimed to identify fundamental biological processes, molecular functions, and cellular components. For GO terms, we consulted non-redundant databases encompassing the three major domains, biological processes, cellular components, and molecular functions. We additionally included pathway annotations from KEGG, Reactome, Panther, and WikiPathway to capture a broad spectrum of potential molecular mechanisms. All GO terms and pathways were prioritized based on their false discovery rate (FDR) values (*p* < 0.01), thereby minimizing the likelihood of false positives and highlighting the most statistically significant findings.

### Benchmarking XAI approaches on MOGONet

To replicate MOGONet on the ROSMAP dataset, we strictly followed the methodology implemented by its authors. The ROSMAP dataset comprises 351 samples, with 169 (48%) classified as normal cases and 182 (52%) as AD cases. The dataset is divided into training and testing sets, with 70% (245 samples) used for training and 30% (106 samples) used for testing. The division is stratified to ensure that Alzheimer’s cases consistently represent 52% of both subsets. Each sample includes three modalities, DNA methylation, mRNA expression, and microRNA expression, each represented by 200 features, which serve as input to the model. Each modality is processed by its respective graph-based model. The initial predictions from these modality-specific graph-based models are then passed to a view correlation discovery network (VCDN), which integrates the information to make a final prediction. MOGONet is trained in two phases: in the first phase, only the graph-based models are trained for 500 epochs using the Adam optimizer with a learning rate of 10^−3^, while the VCDN is not trained. In the second phase, both the graph-based models and the VCDN are trained for 2500 epochs, with the learning rate for the graph-based models halved (5 × 10^−4^) and the VCDN trained with a learning rate of 10^−3^, using the Adam optimizer. All coding was done on a CPU-enabled Google Colab notebook using the PyTorch framework.

### Evaluation of explainability without ground truth

In scenarios where ground truth annotations are unavailable, fidelity, inverse fidelity, and sparsity can be used for evaluation of the explainability. These parameters measure how well the selected components influence the model’s prediction and how concise the explanations are.

Fidelity evaluates how much a selected component (e.g., node, edge, modality, or feature) contributes to the model’s prediction by measuring the performance drop after its removal. Inverse fidelity, on the other hand, measures the performance when only the selected component is retained. Sparsity controls the size of the modified input by penalizing explanations that include too many components, helping to ensure fair comparisons between different scenarios. Ideally, a good explanation yields high fidelity and sparsity, and low inverse fidelity^57^.

2 $${{{\rm{Fidelity}}}}: [f(G)] \, {\mbox{-}} \, [f(G{\backslash} \, g)] 2$$

3 $${{{\rm{Inverse}}}}\; {{{\rm{fidelity}}}}:[f(G)]-[f(g)]$$

4 $${{{\rm{Sparsity}}}}:1-{|g|}{{{\rm{ / }}}}{|G|}$$

In the equations above, $\left[f\left(G\right)\right]$ represents the model’s performance when all components are present, $\left[f\left({G\backslash g}\right)\right]$ denotes the performance when specific components $g$ are removed from the full model $G$, while $\left[f\left(g\right)\right]$ refers to the performance when only those components *g* are retained. |*g*| and |*G*| indicate the number of elements in the selected subset and the full model, respectively.

## Supplementary information

Supplementary information

## Data Availability

The source code and the dataset can be found at https://github.com/alrzsdgh/Graph-Review/.

## References

[1] Sarker, I. H. Deep learning: a comprehensive overview on techniques, taxonomy, applications and research directions. *SN Comput. Sci.* **2**, 420 (2021). 34426802 10.1007/s42979-021-00815-1 PMC8372231

[2] Shoeibi, A. et al. Diagnosis of brain diseases in fusion of neuroimaging modalities using deep learning: a review. *Inf. Fusion* **93**, 85–117 (2023).

[3] Tran, K. A. et al. Deep learning in cancer diagnosis, prognosis and treatment selection. *Genome Med.* **13**, 1–17 (2021). 34579788 10.1186/s13073-021-00968-x PMC8477474

[4] Askr, H. et al. Deep learning in drug discovery: an integrative review and future challenges. *Artif. Intell. Rev.* **56**, 5975–6037 (2023). 36415536 10.1007/s10462-022-10306-1 PMC9669545

[5] Squarcina, L., Villa, F. M., Nobile, M., Grisan, E. & Brambilla, P. Deep learning for the prediction of treatment response in depression. *J. Affect. Disord.* **281**, 618–622 (2021). 33248809 10.1016/j.jad.2020.11.104

[6] Huang, Z. et al. SALMON: survival analysis learning with multi-omics neural networks on breast cancer. *Front. Genet.* **10**, 166 (2019). 30906311 10.3389/fgene.2019.00166 PMC6419526

[7] Singh, A. et al. DIABLO: an integrative approach for identifying key molecular drivers from multi-omics assays,. *Bioinformatics* **35**, 3055–3062 (2019). 30657866 10.1093/bioinformatics/bty1054 PMC6735831

[8] Boehm, K. M., Khosravi, P., Vanguri, R., Gao, J. & Shah, S. P. Harnessing multimodal data integration to advance precision oncology. *Nat. Rev. Cancer* **22**, 114–126 (2022). 34663944 10.1038/s41568-021-00408-3 PMC8810682

[9] Cui, H. et al. Evolving graph convolutional network with transformer for CT segmentation. *Appl. Soft Comput.* **165**, 112069 (2024).

[10] Cui, H. et al. “Co-graph attention reasoning based imaging and clinical features integration for lymph node metastasis prediction.” In *Proc. Medical Image Computing and Computer Assisted Intervention–MICCAI 2021: 24th International Conference*, 657–666 (*Springer,* 2021).

[11] Liu, M. et al. Exploiting geometric features via hierarchical graph pyramid transformer for cancer diagnosis using histopathological images. *IEEE Trans. Med. Imaging* **43**, 2888–2900 (2024). 10.1109/TMI.2024.3381994 38530716

[12] Gaggion, N., Mansilla, L., Mosquera, C., Milone, D. H. & Ferrante, E. Improving anatomical plausibility in medical image segmentation via hybrid graph neural networks: applications to chest X-ray analysis. *IEEE Trans. Med. Imaging* **42**, 546–556 (2022). 10.1109/TMI.2022.3224660 36423313

[13] Xuan, P. et al. Graph triple-attention network for disease-related lncRNA prediction. *IEEE J. Biomed. Health Inform.* **26**, 2839–2849 (2021). 10.1109/JBHI.2021.3130110 34813484

[14] Xuan, P. et al. Meta-path semantic and global-local representation learning enhanced graph convolutional model for disease-related miRNA prediction. *IEEE J. Biomed. Health Inform.* **28**, 4306 –4316 (2024). 10.1109/JBHI.2024.3397003 38709611

[15] La Rosa, M., Fiannaca, A., La Paglia, L. & Urso, A. A graph neural network approach for the analysis of siRNA-target biological networks. *Int. J. Mol. Sci.* **23**, 14211 (2022). 36430688 10.3390/ijms232214211 PMC9696923

[16] Loh, H. W. et al. Application of explainable artificial intelligence for healthcare: a systematic review of the last decade (2011–2022). *Comput. Methods Programs Biomed.* **226**, 107161 (2022). 10.1016/j.cmpb.2022.107161 36228495

[17] Zahedi, R. et al. Deep learning in spatially resolved transcriptomics: a comprehensive technical view. *Brief. Bioinform.* **25**, bbae082 (2024). 10.1093/bib/bbae082 PMC10939360 38483255

[18] Alinejad-Rokny, H. et al. MaxHiC: a robust background correction model to identify biologically relevant chromatin interactions in Hi-C and capture Hi-C experiments. *PLoS Comput. Biol.* **18**, e1010241 (2022). 35749574 10.1371/journal.pcbi.1010241 PMC9262194

[19] Xu, K., Hu, W., Leskovec, J. & Jegelka, S. How powerful are graph neural networks? In *International Conference on Learning Representations.* Available at: https://openreview.net/forum?id=ryGs6iA5Km (2019).

[20] Kipf, T. N. & Welling, M. Semi-supervised classification with graph convolutional networks. In *International Conference on Learning Representations* Available at: https://openreview.net/forum?id=SJU4ayYgl (2017).

[21] Veličković, P. et al. Graph attention networks. In *International Conference on Learning Representations.* Available at: https://openreview.net/forum?id=rJXMpikCZ (2018).

[22] Dwivedi, V. P. & Bresson, X. A generalization of transformer networks to graphs. Preprint at 10.48550/arXiv.2012.09699 (2020).

[23] Yang, H. et al. Interpretable multimodality embedding of cerebral cortex using attention graph network for identifying bipolar disorder. In *Proc.**Medical Image Computing and Computer Assisted Intervention–MICCAI 2019: 22nd International Conference, 799–807*(Springer, 2019).

[24] Qu, G. et al. Ensemble manifold regularized multi-modal graph convolutional network for cognitive ability prediction. *IEEE Trans. Biomed. Eng.* **68**, 3564–3573 (2021). 33974537 10.1109/TBME.2021.3077875

[25] Sebenius, I. et al. Multimodal graph coarsening for interpretable, MRI-based brain graph neural network. In *Proc.** 2021 IEEE 31st International Workshop on Machine Learning for Signal Processing (MLSP)* 1–6 (IEEE, 2021).

[26] Chen, Y. et al. Attention-based node-edge graph convolutional networks for identification of autism spectrum disorder using multi-modal MRI data. In *Proc.** Pattern Recognition and Computer Vision: 4th Chinese Conference, PRCV 2021,*374–385 (Springer, 2021).

[27] Zhou, H., Zhang, Y., Chen, B. Y., Shen, L. & He, L. Sparse interpretation of graph convolutional networks for multi-modal diagnosis of Alzheimer’s disease. In *Proc.**International Conference on Medical Image Computing and Computer-Assisted Intervention*, 469–478 (Springer, 2022). 10.1007/978-3-031-16452-1_45 PMC9942706 36827208

[28] Safai, A. et al. Multimodal brain connectomics-based prediction of Parkinson’s disease using graph attention networks. *Front. Neurosci.* **15**, 741489 (2022). 35280342 10.3389/fnins.2021.741489 PMC8904413

[29] Bi, X. -a et al. Feature aggregation graph convolutional network based on imaging genetic data for diagnosis and pathogeny identification of Alzheimer’s disease. *Brief. Bioinform.* **23**, bbac137 (2022). 35453149 10.1093/bib/bbac137

[30] Kan, X., Kong, Y., Yu, T. & Guo, Y. BraceNet: graph-embedded neural network for brain network analysis. In *Proc.** 2022 IEEE International Conference on Big Data (Big Data)*, 4979–4987 (IEEE, 2022).

[31] Zhou, H., He, L., Chen, B. Y., Shen, L. & Zhang, Y. Multi-modal diagnosis of Alzheimer’s disease using interpretable graph convolutional networks. *IEEE Trans. Med. Imaging* **44**, 142–153 (2024). 10.1109/TMI.2024.3432531 PMC11754532 39042528

[32] Patel, B. et al. Explainable multimodal graph isomorphism network for interpreting sex differences in adolescent neurodevelopment. *Appl. Sci.* **14**, 4144 (2024). 42037656 10.3390/app14104144 PMC13108686

[33] Qu, G., Zhou, Z., Calhoun, V. D., Zhang, A. & Wang, Y.-P. Integrated brain connectivity analysis with fMRI, DTI, and sMRI powered by interpretable graph neural networks. *Med. Image Anal.* **103**, 103570 (2025). 40250104 10.1016/j.media.2025.103570 PMC13178953

[34] Schulte-Sasse, R., Budach, S., Hnisz, D. & Marsico, A. Integration of multiomics data with graph convolutional networks to identify new cancer genes and their associated molecular mechanisms. *Nat. Mach. Intell.* **3**, 513–526 (2021).

[35] Zhang, L. et al. AutoGGN: a gene graph network AutoML tool for multi-omics research. *Artif. Intell. Life Sci.* **1**, 100019 (2021).

[36] Pfeifer, B., Saranti, A. & Holzinger, A. GNN-SubNet: disease subnetwork detection with explainable graph neural networks. *Bioinformatics* **38**, ii120–ii126 (2022). 36124793 10.1093/bioinformatics/btac478

[37] Pfeifer, B., Baniecki, H., Saranti, A., Biecek, P. & Holzinger, A. Graph-guided random forest for gene set selection. Preprint at 10.48550/arXiv.2108.11674 (2021).

[38] Kaczmarek, E. et al. Multi-omic graph transformers for cancer classification and interpretation. In *Pacific Symposium on Biocomputing 2022*, 373–384 (World Scientific, 2021). 34890164

[39] Hou, W. et al. Hybrid graph convolutional network with online masked autoencoder for robust multimodal cancer survival prediction. *IEEE Trans. Med. Imaging* **42**, 2462–2473 (2023). 10.1109/TMI.2023.3253760 37028064

[40] Ma, A. et al. Single-cell biological network inference using a heterogeneous graph transformer. *Nat. Commun.* **14**, 964 (2023). 36810839 10.1038/s41467-023-36559-0 PMC9944243

[41] Keicher, Matthias, et al. Multimodal graph attention network for COVID-19 outcome prediction. *Sci. Rep.* **13**, 19539 (2023). 10.1038/s41598-023-46625-8 PMC10636061 37945590

[42] Wang, T. et al. MOGONET integrates multi-omics data using graph convolutional networks allowing patient classification and biomarker identification. *Nat. Commun.* **12**, 3445 (2021). 34103512 10.1038/s41467-021-23774-w PMC8187432

[43] Chan, Y. H., Wang, C., Soh, W. K. & Rajapakse, J. C. Combining neuroimaging and omics datasets for disease classification using graph neural networks. *Front. Neurosci.* **16**, 866666 (2022). 35677355 10.3389/fnins.2022.866666 PMC9168232

[44] Li, X., Ma, J., Leng, L., Han, M. & Li, M. MoGCN: a multi-omics integration method based on graph convolutional network for cancer subtype analysis,. *Front. Genet.* **13**, 806842 (2022). 35186034 10.3389/fgene.2022.806842 PMC8847688

[45] Bintsi, K.-M., Baltatzis, V., Potamias, R. A., Hammers, A. & Rueckert, D. Multimodal brain age estimation using interpretable adaptive population-graph learning. In *Proc.** International Conference on Medical Image Computing and Computer-Assisted Intervention*, 195–204 (Springer, 2023).

[46] Kazi, A., Farghadani, S., Aganj, I., & Navab, N. IA-GCN: interpretable attention based graph convolutional network for disease prediction. In *Proc.**International Workshop on Machine Learning in Medical Imaging*, 382–392 (Springer, 2023). 10.1007/978-3-031-45673-2_38 PMC10583839 37854585

[47] Bi, X. -a et al. Explainable and programmable hypergraph convolutional network for imaging genetics data fusion. *Inf. Fusion* **100**, 101950 (2023).

[48] Xiao, C., Pham, N., Imel, E. & Luo, X. Patient-GAT: sarcopenia prediction using multi-modal data fusion and weighted graph attention networks. In *Proc. 38th ACM/SIGAPP Symposium on Applied Computing*, 614–617 (ACM, 2023). 10.1145/3555776.3578731 PMC10732263 38125287

[49] Ouyang, D. et al. Integration of multi-omics data using adaptive graph learning and attention mechanism for patient classification and biomarker identification. *Comput. Biol. Med.* **164**, 107303 (2023). 37586201 10.1016/j.compbiomed.2023.107303

[50] Li, Y., Qiao, G., Wang, K. & Wang, G. Drug–target interaction predication via multi-channel graph neural networks. *Brief. Bioinform.* **23**, bbab346 (2022). 34661237 10.1093/bib/bbab346

[51] Lei, B. et al. Alzheimer’s disease diagnosis from multi-modal data via feature inductive learning and dual multilevel graph neural network. *Med. Image Anal.* **97**, 103213 (2024). 38850625 10.1016/j.media.2024.103213

[52] Tang, S. et al. Predicting 30-day all-cause hospital readmission using multimodal spatiotemporal graph neural networks. *IEEE J. Biomed. Health Inform.* **27**, 2071–2082 (2023). 37018684 10.1109/JBHI.2023.3236888 PMC11073780

[53] Ribeiro, M. T., Singh, S. & Guestrin, C. Why should I trust you?” Explaining the predictions of any classifier. In *Proc. 22nd ACM SIGKDD International Conference on Knowledge Discovery and Data Mining*, 1135–1144 (ACM, 2016).

[54] Petsiuk, V., Das, A. & Saenko, K. RISE: randomized input sampling for explanation of black-box models. In *British Machine Vision Conference*. Available at: http://bmvc2018.org/contents/papers/1064.pdf (2018).

[55] Feng, Q. et al. DEGREE: decomposition based explanation for graph neural networks. In *International Conference on Learning Representations* Available at: https://openreview.net/forum?id=Ve0Wth3ptT_ (2022).

[56] Luo, D. et al. Parameterized explainer for graph neural network. *Adv. Neural Inf. Process. Syst.* **33**, 19620–19631 (2020).

[57] Zhang, S., Liu, Y., Shah, N. & Sun, Y. GStarX: explaining graph neural networks with structure-aware cooperative games. *Adv. Neural Inf. Process. Syst.* **35**, 19810–19823 (2022).

[58] Ding, M. et al. VQ-GNN: a universal framework to scale up graph neural networks using vector quantization. *Adv. Neural Inf. Process. Syst.* **34**, 6733–6746 (2021).

[59] Liu, N. et al. Explainable recommender systems via resolving learning representations. In *Proc. 29th ACM International Conference on Information and Knowledge Management*, 895–904 (ACM, 2020).

[60] Ma, J., Cui, P., Kuang, K. Wang, X. & Zhu, W. Disentangled graph convolutional networks. In *Proc.**International Conference on Machine Learning*, 4212–4221 (PMLR, 2019).

[61] Lundberg, S. M. & Lee, S.-I. A unified approach to interpreting model predictions. *Adv. Neural Inf. Process. Syst.* **30**, 4765–4774 (2017).

[62] Simonyan, K., Vedaldi, A. & Zisserman, A. Deep inside convolutional networks: visualising image classification models and saliency maps. Preprint at 10.48550/arXiv.1312.6034 (2013).

[63] Pope, P. E., Kolouri, S., Rostami, M., Martin, C. E., & Hoffmann, H. “Explainability methods for graph convolutional neural networks.” In *Proc. IEEE/CVF Conference on Computer Vision and Pattern Recognition*, 10772–10781 (IEEE, 2019).

[64] Sundararajan, M., Taly, A. & Yan, Q. Axiomatic attribution for deep networks. In *Proc.**International Conference on Machine Learning*, 3319–3328 (PMLR, 2017).

[65] Garson, D. G. Interpreting neural network connection weights. *AI Expert* **6**, 46–51 (1991).

[66] Adebayo, J. et al. Sanity checks for saliency maps. *Adv. Neural Inf. Process. Syst.* **31**, 9525–9536 (2018).

[67] Shrikumar, A., Greenside, P. & Kundaje, A. Learning important features through propagating activation differences. In *Proc.**International Conference on Machine Learning*, 3145−3153 (PMLR, 2017).

[68] Ying, Z., Bourgeois, D., You, J., Zitnik, M. & Leskovec, J. GNNExplainer: generating explanations for graph neural networks. *Adv. Neural Inf. Process. Syst.* **32**, 9240–9251 (2019). PMC7138248 32265580

[69] Cangea, C., Veličković, P., Jovanović, N., Kipf, T. & Liò, P. Towards sparse hierarchical graph classifiers. Preprint at 10.48550/arXiv.1811.01287 (2018).

[70] Hodes, R. J. & Buckholtz, N. Accelerating medicines partnership: Alzheimer’s disease (AMP-AD) knowledge portal aids Alzheimer’s drug discovery through open data sharing. *Expert Opin. Ther. Targets* **20**, 389–391 (2016). 26853544 10.1517/14728222.2016.1135132

[71] Nesvijevskaia, A., Ouillade, S., Guilmin, P. & Zucker, J.-D. The accuracy versus interpretability trade-off in fraud detection model. *Data Policy* **3**, e12 (2021).

[72] Zhang, Z., Tian, R., Sherony, R., Domeyer, J. & Ding, Z. Attention-based interrelation modeling for explainable automated driving,. *IEEE Trans. Intell. Veh.* **8**, 1564–1573 (2022).

[73] Chen, C., Wu, T., Guo, Z. & Cheng, J. Combination of deep neural network with attention mechanism enhances the explainability of protein contact prediction. *Proteins Struct. Funct. Bioinform.* **89**, 697–707 (2021). 10.1002/prot.26052 PMC8089057 33538038

[74] Chefer, H., Gur, S. & Wolf, L. Generic attention-model explainability for interpreting bi-modal and encoder-decoder transformers. In *Proc. IEEE/CVF International Conference on Computer Vision*, 397–406 (IEEE, 2021).

[75] Hakkoum, H., Abnane, I. & Idri, A. Interpretability in the medical field: a systematic mapping and review study. *Appl. Soft Comput.* **117**, 108391 (2022).

[76] Prince, S. J. *Understanding Deep Learning* (MIT Press, 2023).

[77] Rusch, T. K., Bronstein, M. M. & Mishra, S. A survey on oversmoothing in graph neural networks. Preprint at 10.48550/arXiv.2303.10993 (2023).

[78] Topping, J., Di Giovanni, F., Chamberlain, B. P., Dong, X. & Bronstein, M. M. Understanding over-squashing and bottlenecks on graphs via curvature. In *International Conference on Learning Representations* Available at: https://openreview.net/forum?id=7UmjRGzp-A (2022).

[79] Bonabi Mobaraki, E. & Khan, A. A demonstration of interpretability methods for graph neural networks. In *Proc. 6th Joint Workshop on Graph Data Management Experiences and Systems (GRADES) and Network Data Analytics (NDA)*, 1–5 (ACM, 2023).

[80] Gu, J., Ye, J., Uddin, A. & Wang, G. DySTAGE: dynamic graph representation learning for asset pricing via spatio-temporal attention and graph encodings. In *Proc. 5th ACM International Conference on AI in Finance*, 388–396 (ACM, 2024).

[81] Cakmak, E. Jäckle D., Schreck T. & Keim, D. dg2pix: Pixel-Based Visual Analysis of Dynamic Graphs. *2020 Visualization in Data Science (VDS)*, Salt Lake City, UT, USA, pp. 32–41, 10.1109/VDS51726.2020.00008 2020.

[82] Agarwal, C., Queen, O., Lakkaraju, H. & Zitnik, M. Evaluating explainability for graph neural networks. *Sci. Data* **10**, 144 (2023). 36934095 10.1038/s41597-023-01974-x PMC10024712

[83] Yuan, H., Yu, H., Gui, S. & Ji, S. Explainability in graph neural networks: a taxonomic survey,. *IEEE Trans. Pattern Anal. Mach. Intell.* **45**, 5782–5799 (2022). 10.1109/TPAMI.2022.3204236 36063508

[84] Defferrard, M., Bresson, X. & Vandergheynst, P. “Convolutional neural networks on graphs with fast localized spectral filtering.” *Adv. Neural Inf. Process. Syst.* **29**, 3837–3845 (2016).

[85] Bo, D. et al. “A survey on spectral graph neural networks.” Preprint at 10.48550/arXiv.2302.05631 (2023).

[86] R. M. Byrne. “Counterfactuals in explainable artificial intelligence (XAI): evidence from human reasoning.” In *Proc. International Joint Conference on Artificial Intelligence*, 6276–6282 (IJCAI, 2019).

[87] Malandri, L., Mercorio, F., Mezzanzanica, M. & Seveso, A. Model-contrastive explanations through symbolic reasoning. *Decis. Support Syst.* **176**, 114040 (2024).

[88] Yang, X. et al. A large language model for electronic health records. *NPJ Digit. Med.* **5**, 194 (2022). 36572766 10.1038/s41746-022-00742-2 PMC9792464

[89] Taylor, R. et al. “Galactica: a large language model for science.”Preprint at 10.48550/arXiv.2211.09085 (2022).

[90] Thirunavukarasu, A. J. et al. Large language models in medicine. *Nat. Med.* **29**, 1930–1940 (2023). 37460753 10.1038/s41591-023-02448-8

[91] Zhu, Y. et al. LLMs for knowledge graph construction and reasoning: recent capabilities and future opportunities. *World Wide Web* **27**, 58 (2024).

[92] Yan, Y., Hou, Y., Xiao, Y., Zhang, R. & Wang, Q. “KNOWNET: guided health information seeking from LLMs via knowledge graph integration.” *IEEE Trans. Visualization Comput. Graphics* **31**, 547–557 (2024). 10.1109/TVCG.2024.3456364 PMC11875928 39255106

[93] Gao, Y. et al. Leveraging medical knowledge graphs into large language models for diagnosis prediction: design and application study. *JMIR AI* **4**, e58670 (2025). 39993309 10.2196/58670 PMC11894347

[94] He, X. et al. “Harnessing explanations: LLM-to-LM interpreter for enhanced text-attributed graph representation learning.” In *International Conference on Learning Representations* Available at: https://openreview.net/forum?id=RXFVcynVe1 (2024).

[95] Zhang, X. et al. “From Redundancy to Relevance: Information Flow in {LVLM}s Across Reasoning Tasks.” In *Proceedings of the 2025 Conference of the Nations of the Americas Chapter of the Association for Computational Linguistics: Human Language Technologies* Available at: https://aclanthology.org/2025.naacl-long.115/ (2025).

[96] Singhal, K. et al. Large language models encode clinical knowledge. *Nature* **620**, 172–180 (2023). 37438534 10.1038/s41586-023-06291-2 PMC10396962

[97] Tamkin, A., Brundage, M., Clark, J. & Ganguli, D. “Understanding the capabilities, limitations, and societal impact of large language models.” Preprint at 10.48550/arXiv.2102.02503 (2021).

[98] Huang, L. et al. “A survey on hallucination in large language models: principles, taxonomy, challenges, and open questions.” *ACM Trans. Inf. Syst*. **43**, 1–55 (2025).

[99] Gallegos, I. O. et al. “Bias and fairness in large language models: a survey.” *Comput. Linguistics* **50**, 1097–1179 (2024).

[100] Wang, J., Vasaikar, S., Shi, Z., Greer, M. & Zhang, B. WebGestalt 2017: a more comprehensive, powerful, flexible and interactive gene set enrichment analysis toolkit. *Nucleic Acids Res.* **45**, W130–W137 (2017). 28472511 10.1093/nar/gkx356 PMC5570149

