# Supplementary material for: Interpretable graph-based models on multimodal biomedical data integration: a technical review and benchmarking
Source: Nat Commun. 2026 Jun 16;17:5405. doi: 10.1038/s41467-026-74126-5 (PMC13280480; doi:10.1038/s41467-026-74126-5)
Supplement: Supplementary file 1 — Supplementary information [file 41467_2026_74126_MOESM1_ESM.pdf]

Interpretable graph-based models on multimodal biomedical data integration: A technical review and benchmarking

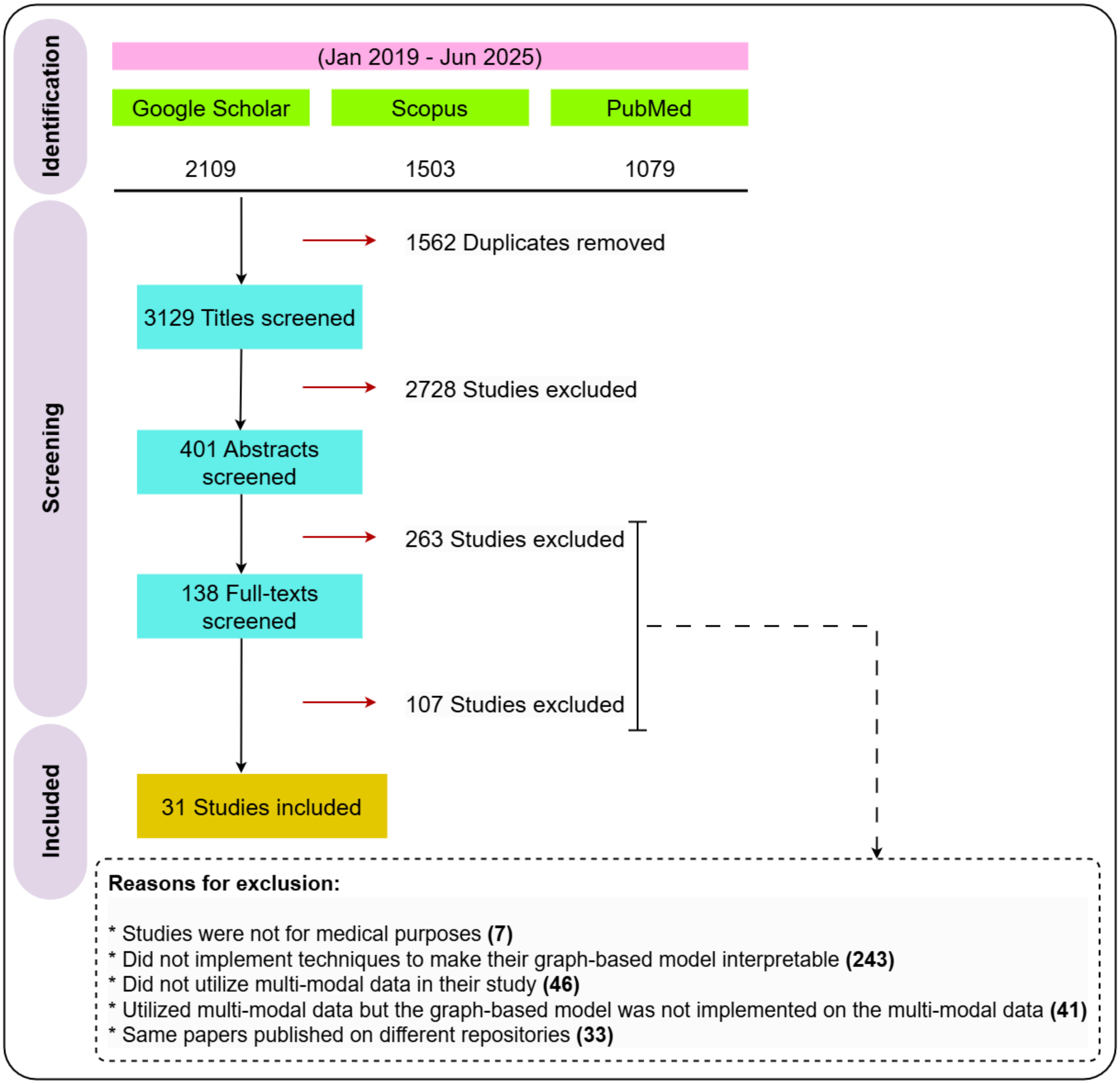

Figure S1. The PRISMA diagram illustrates the process of retrieving related papers for this review study. PRISMA: Preferred Reporting Items for Systematic Reviews and Meta-Analyses.

Table S1. Detailed description of the papers reviewed in this study.

Study	Objective	Task Level	Modalities Used	Graph Construction	Interpretability Objective	Interpretability Technique	Interpretability Mode
Yang et al. [1]	Classifying bipolar disorder (manic depression) vs healthy cases	Graph	sMRI and fMRI	**Nodes:** Each patient's brain ROIs<br>**Edges:** Fully connected graph where each edge's weight is calculated using Pearson correlation between node's features.<br>**Feature vector:** Each node's feature vector is generated using combination of features from fMRI and sMRI.	Importance of each modality<br><br>Importance of each feature and their coactivation	Replacing modalities' features with dummy variable<br><br>Attention within model structure	Post processing<br><br>Interpretable Model
Keicher et al. [2]	To predict COVID-19 outcome	Node	CT, radiomic, and meta data	**Nodes:** Each patient<br>**Edges:** Distance between nodes are calculated using weighted Minkowski on the combination of radiomic and meta data features and each node is connected to its k nearest neighbors.<br>**Feature vector:** A combination of features from all modalities.	Which neighbor patients have more impact on a specific patient prediction (proper for pandemic situations)	Attention in GAT	Interpretable Model
Qu et al. [3]	To predict various physiological phenotypes	Graph	emoid-fMRI and nback-fMRI	For each patient, two graphs based on each modality are constructed.<br>**Nodes:** Brain ROIs.<br>**Edges in each graph:** Pearson correlation between nodes is calculated and the k-nearest neighbor approach implemented subsequently.<br>**Feature vector:** Features from each modality for their corresponding graph.	Importance of each modality<br><br>Importance of each node (brain's ROI)<br><br>Importance of each edge (relationship between ROIs)	Eliminating modalities<br><br>Grad – RAM<br><br>Edge masking	Post processing<br><br>Post processing<br><br>Post processing

Study	Objective	Task Level	Modalities Used	Graph Construction	Interpretability Objective	Interpretability Technique	Interpretability Mode
Wang et al. [4]	Biomedical classification	Node	mRNA expression, DNA methylation, and microRNA expression data	For each patient, three graphs are constructed based on each modality.<br>**Node:** Each patient<br>**Edges in each graph:** Cosine similarity between each node is calculated and edges with value higher a certain threshold are maintained.<br>**Feature vector:** Features from each modality for their corresponding graph.	Importance of each modality	Eliminating modalities	Post processing
Schulte-Sasse [5]	Different cancer gene prediction	Node	Mutations, DNA methylation and gene expression	For each cancer, a separate semi-labeled graph is constructed.<br>**Nodes:** genes<br>**Edges:** gene – gene interactions<br>**Feature vectors:** A combination of features from all modalities.	Importance of each modality	Eliminating modalities	Post processing
Sebenius [6]	To detect Schizophrenia patients	Graph	fMRI and Morphometric similarity	For each patient, two graphs are constructed based on each modality<br>**Nodes:** Each patient's brain ROIs<br>**Edges**: Pairwise correlation based on each graph's node features is calculated and edges with values higher than a certain threshold are maintained.<br>**Feature vector**: Features from each modality for their corresponding graph.	Importance of each modality<br><br>Importance of each ROI	Eliminating modalities<br><br>Top – k ranking	Post processing<br><br>Post processing
Zhang et al. [7]	To classify breast cancer subtypes	Graph	Protein and gene expression profiles	**Nodes:** Protein<br>**Edges:** Protein – protein interactions<br>**Feature vector:** A combination of features derived from different modalities.	Importance of each feature	SHAP	Post processing

Study	Objective	Task Level	Modalities Used	Graph Construction	Interpretability Objective	Interpretability Technique	Interpretability Mode
Chen et al. [8]	To detect autism spectrum disorder	Graph	sMRI and rsfMRI	**Nodes:** Brain ROIs<br>**Edges:** Fully connected graph where each edge's weight is calculated using Pearson correlation between node's based on their fMRI features.<br>**Feature Vector:** A combination of features from both modalities.	Importance of each modality<br><br>Importance of each feature	Eliminating modalities<br><br>Gradient saliency map	Post processing<br><br>Post processing
Pfeifer et al. [9]	Cancer patient classification	Graph	mRNA gene expression and DNA methylation	**Nodes:** Protein<br>**Edges:** Protein – protein interactions<br>**Feature vector:** A combination of features from both modalities.	The most important subgraph in each patient's graph	GNNExplainer	Post processing
Zhou et al. [10]	Alzheimer disease classification	Graph	Structural MRI (VBM-MRI), fluorodeoxyglucose PET (FDG-PET), and 18-F florbetapir PET (AV45-PET)	**Nodes:** Each patient's brain ROIs<br>**Edges:** Gaussian similarity function of Euclidean distances of each node based on their features is calculated and each node is connected to its k nearest neighbors.<br>**Feature vector:** A combination of features derived from each modality.	Importance of each ROI	Graph masking	Post processing

Study	Objective	Task Level	Modalities Used	Graph Construction	Interpretability Objective	Interpretability Technique	Interpretability Mode
Chan et al. [11]	Parkinson disease classification	Node	Image (fMRI, DTI)<br><br>omics (SNP, sncRNA, miRNA, RNA sequencing and DNA Methylation)	Two graphs are constructed based on imagery and omics features<br>**Nodes:** Patients<br>**Edges:** Fully connected with each edge's weight calculated based on Pearson correlation between feature nodes.<br>**Feature vector:** imagery features are fed to its corresponding graph and after processing by GCN, the outputs are fed as feature to omics graphs.	Importance of each modality	Eliminating modalities and attention mechanism	Post processing
Safai et al. [12]	Parkinson disease classification	Graph	fMRI, TI weighted MRI, DWI	**Nodes:** Each patient's brain ROIs<br>**Edges:** Conditional probability based on DWI features and considering a certain threshold<br>**Feature vector:** A combination of features derived from all modalities.	Importance of each modality<br><br>Importance of each feature<br><br>Importance of each ROI	Eliminating modalities<br><br>Gradient saliency map<br><br>Attention mechanism	Post processing<br><br>Post processing<br><br>Interpretable model
Li et al. [13]	To classify breast cancer patients into their subtypes	Node	Copy number variation (CNV) mRNA, and reverse phase protein array data (RPPA)	**Nodes:** Patients<br>**Edges:** Similarity of nodes is calculated based on Euclidean distance and subsequently, k-nearest method is applied to maintain more important edges.<br>**Feature vector:** A combination of features derived from all modalities.	Importance of each modality	Sensitivity analysis	Post processing

Study	Objective	Task Level	Modalities Used	Graph Construction	Interpretability Objective	Interpretability Technique	Interpretability Mode
Bi et al. [14]	Alzheimer disease classification	Graph	fMRI and SNP	**Nodes:** Each patient's brain ROIs and genes **Edges:** Two types of edges are constructed. First called 'weights' based on Pearson correlation between nodes and considering a certain threshold. The second one is 'similarity-based' which is draw when two nodes share a certain number of neighbors. **Feature vector:** Features from fMRI are used for ROIs and features from SNP are utilized for genes.	Importance of each modality<br><br>Importance of each node (brain ROI) and each feature	Eliminating modalities<br><br>Gradient saliency map	Post processing<br><br>Post processing
Li et al. [15]	To find targets for drugs	Node	Protein and drug	Two graphs are constructed (topology graph and feature graph) **Nodes:** Drug – protein pairs **Edges:** For topology graph, two nodes are linked if they share a drug or protein. For feature graph, cosine similarity between nodes is calculated and each node is connected to its k nearest neighbors. **Feature vector:** A combination of feature derived from proteins and drugs.	Importance of other nodes on each node's prediction	Attention in GAT	Interpretable model

Study	Objective	Task Level	Modalities Used	Graph Construction	Interpretability Objective	Interpretability Technique	Interpretability Mode
Pfeifer et al. [16]	To predict the survival status of patients suffering kidney cancer	Graph	Gene expression (mRNA) and DNA Methylation	One graph for each patient **Nodes:** Proteins **Edges:** Protein-protein interactions **Feature vector:** features derived from each modality	Importance of each modality	Comparing the performance of the decision tree generated by each specific module	Interpretable Model
					Importance of each feature	Gini impurity index (a standard tree-based importance measures)	Interpretable Model
					Importance of each node (protein)	SHAP	Post Processing
Kaczmarek et al. [17]	To classify cancer	Graph	microRNA and mRNA	One graph for each patient **Nodes:** mRNA and miRNA molecules **Edges:** Edges are formed between nodes based on miRNA-mRNA targeting using TargetScan. **Feature vector:** respective expression of the molecule in each patient	Modality importance	modality elimination	Post Processing
					Which nodes are more important to classify each cancer	Graph Transformer	Interpretable Model
					Which mRNA and miRNA interaction plays crucial role in each cancer classification	Graph Transformer	Interpretable Model
Kan et al. [18]	To predict patients' gender	Graph	fMRI and DTI	**Nodes:** Each patient's brain ROI **Edges:** Nodes are connected if they are correlated via fMRI (pairwise correlation) or DTI (tractography algorithm) **Feature vector:** features derived from each modality	Importance of each feature Importance of each brain ROI	weights values in the model	Interpretable Model

Study	Objective	Task Level	Modalities Used	Graph Construction	Interpretability Objective	Interpretability Technique	Interpretability Mode
Bintsi et al. [19]	Brain aging prediction	Node	Imaging (structural MRI and diffusion weighted MRI)<br><br>clinical data	**Nodes**: Patients<br>**Edges:** Weighted features are generated by feeding Imagery and non-imagery features to an MLP. By calculating the probability of connection between two nodes and a modified version of KNN method, edges are draw.<br>**Feature vector:** Feature derived from imagery data	Importance of each modality<br><br>Importance of each feature	Eliminating modalities<br><br>Attention mechanism	Post processing<br><br>Interpretable model
Kazi et al. [20]	To detect patients with Alzheimer's disease<br><br>To predict patient's age and sex	Node	Cognitive tests, MRI ROIs measures, PET imaging, DTI ROI measures, demographics, etc.<br><br>MRI and fMRI images	Automatic graph construction through training process<br>**Nodes:** Patients<br>**Edges:** edge values are calculated using Euclidean distance between nodes based on their features and edges with values higher than a threshold (defined through training process) are maintained.<br>**Feature vector:** Features derived from mentioned modalities for each task	Feature importance	Attention mechanism	Interpretable Model

Study	Objective	Task Level	Modalities Used	Graph Construction	Interpretability Objective	Interpretability Technique	Interpretability Mode
Tang et al. [21]	To predict 30-day all-cause hospital readmission rate	Node	EHR data and chest radiograph	Two graphs are constructed for each modality **Nodes:** each node is a hospital admission **Edges:** for both graph, edges are calculated based on the Euclidean distance between node's EHR features and values bellow a threshold are eliminated **Feature vector:** features extracted from each modality	Importance of each feature and neighboring nodes	GNNExplainer	Post processing
Bi et al. [22]	To detect Alzheimer's disease	Node	fMRI and SNP genetic data	**Nodes:** Patients **Edges:** two nodes remain connected if their connection strength ranks among the top k edge values calculated based on the Euclidean distance of fMRI or SNP data. Then the remained edge values are calculated based on Pearson correlation between connected nodes**.** **Feature vector:** concatenation of the features derived from each modality	Importance of each feature	graph convolutional filter weights	Interpretable Model

Study	Objective	Task Level	Modalities Used	Graph Construction	Interpretability Objective	Interpretability Technique	Interpretability Mode
Huo et al. [23]	Cancer survival prediction	Graph	Pathological slide, clinical records and genomic profile	One graph is generated for each modality<br>**Nodes:** image sections in pathological graph, demographic and medical reports for clinical graph, and five genomic embeddings using GSEA for genomic graph<br>**Edges:** clinical and genomic graphs are fully connected while nodes in pathological graph are connected to their adjacent spatial neighbors<br>**Feature vector:** features derived by feeding pathological slides to KimiaNet for pathological graph, one-hot encodings for clinical graph, and genomic embeddings for genomic graph	Importance of different regions in pathological slides and features of clinical records<br><br>Importance of each feature in genomic profile	Attention mechanism<br><br>Integrated gradient analysis	Interpretable Model<br><br>Post processing
Xiao et al. [24]	To predict sarcopenia	Node	Demographics and Lab data	**Nodes:** Patients<br>**Edges:** patient similarity is calculated based on the demographic's features (cosine similarity) Lab features (DTW). These similarities are then combined. Each node is connected to its m neighbors with highest edge value.<br>**Feature vector:** features derived from each modality	Contribution of each patient on other patient's prediction	Attention in GAT	Interpretable Model
Ouyang et al. [25]	Disease classification	Node	mRNA expression, DNA methylation and miRNA expression	for each modality, a graph is constructed dynamically through model training<br>**Nodes:** Patients<br>**Edges:** are calculated based on weighted cosine similarity considering node's features<br>**Feature vector:** for each graph, features derived from each modality	Modality importance<br><br>Feature importance	Attention mechanism<br><br>Inner product regularization	Interpretable Model<br><br>Interpretable Model

Study	Objective	Task Level	Modalities Used	Graph Construction	Interpretability Objective	Interpretability Technique	Interpretability Mode
Ma et al. [26]	Learns relations among cells and genes	Node	Genes, proteins, or peak regions	**Nodes:** Genes and cells<br>**Edges:** a gene node is connected to a cell node if the gene exists in the cell<br>**Feature vector:** features derived from each modality	Importance of genes to a specific cell	Attention mechanism	Interpretable Model
Lei et al. [27]	Alzheimer Disease Diagnosis	Graph	Protein, SNP, MRI, and Clinical Data	**Nodes:** Features extracted from each modality.<br>**Edges:** All nodes are connected, and Pearson correlation is used as the weights for edges.<br>**Feature vector:** A specific feature representation module is proposed to find features for each modality in the same feature space.	Finding important protein, SNP, and brain ROI	gradient saliency map	Post processing
Zhou et al. [28]	Alzheimer Disease Diagnosis	Graph	MRI and PET	**Nodes:** Brain ROIs<br>**Edges:** Gaussian similarity function based on Euclidean distance is calculated for each pair of nodes and K-nearest neighbor is used to keep the top K edges.<br>**Feature vector:** features extracted from modalities	Importance of each ROI<br><br>Importance of edges between ROIs	Applying feature importance probability<br><br>Applying edge importance probability	Interpretable Model<br><br>Interpretable Model
Zhang et al. [29]	Cancer diagnosis	Graph	WSI and genomic data	Two graphs are generated for WSI and genomics data, and then these subgraphs are merged to form a bigger heterogenous graph<br><br>**Nodes:** WSI non-overlapping patches for WSI subgraph and biological pathways for genomics one.<br>**Edges:** in WSI subgraph, nodes are connected to their k-nearest patches. In genomics subgraph, nodes are connected to the other k nodes which have the greatest	Importance of each section in WSI<br><br>importance of genomic data (pathways)	Attention weights<br><br>SHAP	Interpretable Model<br><br>Post process

Study	Objective	Task Level	Modalities Used	Graph Construction	Interpretability Objective	Interpretability Technique	Interpretability Mode
				number of common genes. for the heterogenous graph, each node from one modality is connected to all nodes from the other one.<br>**Feature vector:** for WSI subgraph, image-based features extracted by resnet50. for genomic graph, sequencing data of gene contained in each pathway node.			
Patel et al. [30]	Interpreting Sex Differences in Adolescent Neurodevelopment	Graph	emoid-fMRI and nback-fMRI	**Nodes:** Brain ROIs<br>**Edges:** Nodes are connected considering the Pearson correlation of blood oxygenation level-dependent (BOLD) between ROIs. Each edge is then assigned with a weight equal to the cosine similarity between the nodes.<br>**Feature vector: F**eatures extracted from each modality and then concatenated.	Importance of each feature	GNNExplainer	Post processing
Qu et al. [31]	Analyzing cognitive functions throughout youth	Graph	rs-fMRI, DTI, and sMRI	**Nodes:** Brain ROIs<br>**Edges:** Features of each ROI are connected to their top K-nearest neighbors considering Spearman rank correlation.<br>**Feature vector: F**eatures extracted from each modality	Importance of each edge (relationship between ROIs)<br><br>Importance of each feature	Edge Masking<br><br>Grad-RAM and Grad-CAM	Post processing<br><br>Post processing

Table S2. Summary of graph-based modeling approaches and the studies in which they were implemented.

Model	Studies
DNN	Kan et al. [18]
GNN	Sebenius [6], Tang et al. [21]
GIN	Pfeifer et al. [9], Patel et al. [30]
GCN	Qu et al. [3], Wang et al. [4], Schulte-Sasse [5], Zhang et al. [7], Chen et al. [8], Zhou et al. [10], Chan et al. [11], Li et al. [13], Bi et al. [14], Bintsi et al. [19], Kazi et al. [20], Bi et al. [22], Huo et al. [23], Ouyang et al. [25], Zhou et al. [28], Zhang et al. [29], Qu et al. [31]
GAT	Yang et al. [1], Keicher et al. [2], Safai et al. [12], Li et al. [15], Xiao et al. [24], Zhang et al. [29]
GTN	Kaczmarek et al. [17], Ma et al. [26], Lei et al. [27]

Table S3. A summary of explainability techniques and their applications in reviewed studies based on the categories we introduced in our study.

Explainability Category	Explainability Technique	Studies
Category I	Modality Elimination	Yang et al. [1], Qu et al. [3], Wang et al. [4], Schulte-Sasse [5], Sebenius [6], Chen et al. [8], Chan et al. [11], Safai et al. [12], Bi et al. [14], Kaczmarek et al. [17], Bintsi et al. [19]
Category II	Gradient Saliency Map	Chen et al. [8], Safai et al. [12], Bi et al. [14], Lei et al. [27]
	Integrated Gradient Analysis	Huo et al. [23]
	Grad-RAM	Qu et al. [3], Qu et al. [31]
	Sensitivity Analysis	Li et al. [13]
	SHAP	Zhang et al. [7], Pfeifer et al. [16], Zhang et al. [29]
Category III	GNNExplainer	Pfeifer et al. [9], Tang et al. [21], Patel et al. [30]

Explainability Category	Explainability Technique	Studies
	Top-k Ranking	Sebenius [6]
	Graph Masking	Zhou et al. [10]
	Edge Masking	Qu et al. [3], Qu et al. [31]
Category IV	Interpretable Model	Yang et al. [1], Keicher et al. [2], Safai et al. [12], Li et al. [15], Pfeifer et al. [16], Kaczmarek et al. [17], Kan et al. [18], Bintsi et al. [19], Kazi et al. [20], Bi et al. [22], Huo et al. [23], Xiao et al. [24], Ouyang et al. [25], Ma et al. [26], Zhou et al. [28], Zhang et al. [29]

Table S4. The summary of various model-agnostic XAI techniques employed in the surveyed studies alongside their respective formulas. XAI: eXplainable Artificial Intelligence, SHAP: SHapley Additive exPlanations, ROI: Regions Of Interest.

XAI Technique	Explanation	Formula	Denotation
Gradient saliency map	A method to determine the importance of each entity in a model by calculating the gradient of the model's prediction with respect to that specific entity.	$\frac{\partial \hat{y}}{\partial E}$	$\hat{y}$: The score vector produced by the model., E: The specific value associated with an entity in the model, which could refer to a node, its features, or edges.
Integrated gradient [32]	A refined version of the gradient saliency map, mitigates sensitivity	$(x_i - \acute{x}_i) \int_{\alpha=0}^{1} \frac{\partial F(\acute{x} + \alpha(x - \acute{x}))}{\partial x_i} d\alpha$	x: real input, $\acute{x}$: baseline (Black image for imagery, zero embedding vector for text), F: model's function

	breakdown commonly observed in basic gradient saliency maps.		
Grad-RAM [3]	A method to track the gradient of the model with respect to the prediction value	$\frac{1}{NC}ReLU\left(\sum_{n=1\,C=1}^{NC}\frac{\partial\hat{y}_n}{\partial E}\,E\right)$	N: Number of samples, C: number of feature channels, $\hat{y}_n$: the label for the nth subject, E: The specific value associated with an entity in the model, which could refer to a node, its features, or edges.
SHAP [33]	A method that integrates multiple additive XAI techniques, offering feature importance explanations that are more intuitive for humans.	$\sum_{\acute{z}\subseteq\acute{x}}\frac{\lvert\acute{z}\rvert!\,(M-\lvert\acute{z}\rvert-1)!}{M!}[f_x(\acute{z})-f_x(\acute{z}\backslash i)]$	$\lvert\acute{z}\rvert$: the number of non-zero entries in $\acute{z}$, $\acute{x}$: simplified input corresponds to the original input x, M: the number of simplified input features, f_x: model's function, $\backslash i$: excluding feature i values from the input.
Sensitivity analysis [34]	A method employed to quantitatively describe the significance of input variables in neural networks.	$\sigma_i\times\sum_{j=1}^{L}\lvert W_{ij}\rvert$	σ_i: standard deviation of a feature x_i, W_{ij}: the connection weight of the input nodes to the output nodes, L: the number of nodes in the next layer

Table S5. Summary of various graph-based XAI techniques employed in the surveyed studies alongside their respective formulas.

XAI Technique	Explanation	Formula	Denotation
Graph masking	A method systematically eliminates certain parts of the graph structure along with their corresponding feature vectors to identify the most crucial ones	$G_s = A \odot P_A, X_s = X \odot P_X$	G_s: Target important subgraph of the original graph, X_s: Target important subset of feature vector X, A: Adjacency matrix, P_A: Learnable edge importance probability, P_X: Learnable feature importance probability
GNNExplainer [35]	A method similar to graph masking technique except it focuses only on the construction graph to find the most important subgraph instead of analyzing the whole graph	$-\sum_{c=1}^{C} \mathbb{1}[y=c] \log P_\Phi(Y = y \| G = A_c \odot \sigma(M), X = X_s \odot F)$	c: a specific class among a total number of C classes, A_c: Computation graph's adjacency matrix, σ: the sigmoid function, M: Learnable graph mask, F: Learnable feature mask
Top-k pooling [36]	A method to calculate an importance score for each node in a graph	$\delta((\|p\|)^{-1} X p)$	δ: a non-linear function, p: a learnable vector, X: input samples, $\|\cdot\|$: L_2 *norm*

Table S6. Summary of disease areas investigated across the reviewed studies, detailing the specific conditions analyzed and the corresponding graph-based models employed for each.

Disease	Studies
Bipolar disorder	Yang et al. [1]
COVID-19	Keicher et al. [2]

Cancer	Schulte-Sasse [5], Zhang et al. [7], Pfeifer et al. [9], Li et al. [13], Pfeifer et al. [16], Kaczmarek et al. [17], Huo et al. [23], Ouyang et al. [25], Wang et al. [4], Zhang et al. [29]
Schizophrenia	Sebenius [6]
Autism	Chen et al. [8]
Alzheimer	Zhou et al. [10], Bi et al. [14], Kazi et al. [20], Bi et al. [22], Wang et al. [4], Lei et al. [27], Zhou et al. [28]
Parkinson	Chan et al. [11], Safai et al. [12]
Sarcopenia	Xiao et al. [24]

Table S7. Summary of the diverse biomedical data types (e.g., transcriptomics, genomics, clinical records, imaging) used across the reviewed studies, along with the corresponding references.

Data type	Studies
MRI	Safai et al. [12], Bintsi et al. [19], Kazi et al. [20], Lei et al. [27], Zhou et al. [28]
sMRI	Yang et al. [1], Sebenius [6], Chen et al. [8], Zhou et al. [10], Qu et al. [31]
fMRI	Yang et al. [1], Qu et al. [3], Chen et al. [8], Chan et al. [11], Safai et al. [12], Bi et al. [14], Kan et al. [18], Kazi et al. [20], Bi et al. [22], Patel et al. [30], Qu et al. [31]
CT scans	Keicher et al. [2]
Radiography	Keicher et al. [2], Tang et al. [21]
mRNA	Wang et al. [4], Pfeifer et al. [9], Li et al. [13], Pfeifer et al. [16], Kaczmarek et al. [17], Ouyang et al. [25]
DNA	Wang et al. [4] , Schulte-Sasse [5], Pfeifer et al. [9], , Chan et al. [11], Pfeifer et al. [16], Ouyang et al. [25]
microRNA	Wang et al. [4], , Chan et al. [11], Kaczmarek et al. [17], Ouyang et al. [25]
Gene expression	Schulte-Sasse [5], Zhang et al. [7], Pfeifer et al. [9], Pfeifer et al. [16], Ma et al. [26], Zhang et al. [29]
Protein expression	Zhang et al. [7], Li et al. [15], Ma et al. [26], Lei et al. [27]
Positron Emission Tomography (PET)	Zhou et al. [10], Kazi et al. [20], Zhou et al. [28]

Data type	Studies
Single Nucleotide Polymorphism (SNP)	Chan et al. [11], Bi et al. [14], Bi et al. [22], Lei et al. [27]
Clinical data	Bintsi et al. [19], Huo et al. [23], Xiao et al. [24] , Lei et al. [27]
Electrical health records (EHR)	Tang et al. [21]
Diffusion tensor imaging (DTI)	Kan et al. [18], Kazi et al. [20], Chan et al. [11], Qu et al. [31]
Diffusion weighted imaging (DWI)	Safai et al. [12]
Whole Slide Imaging (WSI)	Zhang et al. [29]

Table S8. Some examples of the queries used in our study to identify the papers.

Example 1	Explainable graph networks on multimodal medical data
Example 2	Interpretable graph networks on integrated healthcare data
Example 3	Explainable graph networks on fusion genetic data
Example 4	Transparent graph networks on heterogenous pathology data
Example 5	Transparent graph networks on cross-modal health records
Example 6	Explainable graph networks on multi-source omics data
Example 7	Interpretable graph networks on multimodal radiology data
Example 8	Transparent graph networks on integrated fMRI data

Supplementary References

[1] H. Yang *et al.*, "Interpretable multimodality embedding of cerebral cortex using attention graph network for identifying bipolar disorder," in *Medical Image Computing and Computer Assisted Intervention–MICCAI 2019: 22nd International Conference, Shenzhen, China, October 13–17, 2019, Proceedings, Part III 22*, 2019: Springer, pp. 799-807.

[2] M. Keicher *et al.*, "U-gat: Multimodal graph attention network for covid-19 outcome prediction," *arXiv preprint arXiv:2108.00860,* 2021.

[3] G. Qu *et al.*, "Ensemble manifold regularized multi-modal graph convolutional network for cognitive ability prediction," *IEEE Transactions on Biomedical Engineering,* vol. 68, no. 12, pp. 3564-3573, 2021.

[4] T. Wang *et al.*, "MOGONET integrates multi-omics data using graph convolutional networks allowing patient classification and biomarker identification," *Nature communications,* vol. 12, no. 1, p. 3445, 2021.

[5] R. Schulte-Sasse, S. Budach, D. Hnisz, and A. Marsico, "Integration of multiomics data with graph convolutional networks to identify new cancer genes and their associated molecular mechanisms," *Nature Machine Intelligence,* vol. 3, no. 6, pp. 513-526, 2021.

[6] I. Sebenius, A. Campbell, S. E. Morgan, E. T. Bullmore, and P. Liò, "Multimodal graph coarsening for interpretable, MRI-based brain graph neural network," in *2021 IEEE 31st International Workshop on Machine Learning for Signal Processing (MLSP)*, 2021: IEEE, pp. 1-6.

[7] L. Zhang *et al.*, "AutoGGN: a gene graph network AutoML tool for multi-omics research," *Artificial Intelligence in the Life Sciences,* vol. 1, p. 100019, 2021.

[8] Y. Chen *et al.*, "Attention-based node-edge graph convolutional networks for identification of autism spectrum disorder using multi-modal mri data," in *Pattern Recognition and Computer Vision: 4th Chinese Conference, PRCV 2021, Beijing, China, October 29–November 1, 2021, Proceedings, Part III 4*, 2021: Springer, pp. 374-385.

[9] B. Pfeifer, A. Saranti, and A. Holzinger, "GNN-SubNet: disease subnetwork detection with explainable graph neural networks," *Bioinformatics,* vol. 38, no. Supplement_2, pp. ii120-ii126, 2022.

[10] H. Zhou, Y. Zhang, B. Y. Chen, L. Shen, and L. He, "Sparse interpretation of graph convolutional networks for multi-modal diagnosis of alzheimer's disease," in *International Conference on Medical Image Computing and Computer-Assisted Intervention*, 2022: Springer, pp. 469-478.

[11] Y. H. Chan, C. Wang, W. K. Soh, and J. C. Rajapakse, "Combining neuroimaging and omics datasets for disease classification using graph neural networks," *Frontiers in Neuroscience,* vol. 16, p. 866666, 2022.

[12] A. Safai *et al.*, "Multimodal brain connectomics-based prediction of Parkinson's disease using graph attention networks," *Frontiers in Neuroscience,* vol. 15, p. 741489, 2022.

[13] X. Li, J. Ma, L. Leng, M. Han, and M. Li, "MoGCN: a multi-omics integration method based on graph convolutional network for cancer subtype analysis," *Frontiers in Genetics,* vol. 13, p. 806842, 2022.

[14] X.-a. Bi *et al.*, "Feature aggregation graph convolutional network based on imaging genetic data for diagnosis and pathogeny identification of Alzheimer's disease," *Briefings in Bioinformatics,* vol. 23, no. 3, p. bbac137, 2022.

[15] Y. Li, G. Qiao, K. Wang, and G. Wang, "Drug–target interaction predication via multi-channel graph neural networks," *Briefings in Bioinformatics,* vol. 23, no. 1, p. bbab346, 2022.

[16] B. Pfeifer, H. Baniecki, A. Saranti, P. Biecek, and A. Holzinger, "Graph-guided random forest for gene set selection," *arXiv preprint arXiv:2108.11674,* 2021.

[17] E. Kaczmarek, A. Jamzad, T. Imtiaz, J. Nanayakkara, N. Renwick, and P. Mousavi, "Multi-omic graph transformers for cancer classification and interpretation," in *PACIFIC SYMPOSIUM ON BIOCOMPUTING 2022*, 2021: World Scientific, pp. 373-384.

[18] X. Kan, Y. Kong, T. Yu, and Y. Guo, "Bracenet: Graph-embedded neural network for brain network analysis," in *2022 IEEE International Conference on Big Data (Big Data)*, 2022: IEEE, pp. 4979-4987.

[19] K.-M. Bintsi, V. Baltatzis, R. A. Potamias, A. Hammers, and D. Rueckert, "Multimodal brain age estimation using interpretable adaptive population-graph learning," in *International Conference on Medical Image Computing and Computer-Assisted Intervention*, 2023: Springer, pp. 195-204.

[20] A. Kazi, S. Farghadani, I. Aganj, and N. Navab, "Ia-gcn: Interpretable attention based graph convolutional network for disease prediction," in *International Workshop on Machine Learning in Medical Imaging*, 2023: Springer, pp. 382-392.

[21] S. Tang *et al.*, "Predicting 30-day all-cause hospital readmission using multimodal spatiotemporal graph neural networks," *IEEE Journal of Biomedical and Health Informatics,* vol. 27, no. 4, pp. 2071-2082, 2023.

[22] X.-a. Bi, S. Luo, S. Jiang, Y. Wang, Z. Xing, and L. Xu, "Explainable and programmable hypergraph convolutional network for imaging genetics data fusion," *Information Fusion,* vol. 100, p. 101950, 2023.

[23] W. Hou, C. Lin, L. Yu, J. Qin, R. Yu, and L. Wang, "Hybrid graph convolutional network with online masked autoencoder for robust multimodal cancer survival prediction," *IEEE Transactions on Medical Imaging,* 2023.

[24] C. Xiao, N. Pham, E. Imel, and X. Luo, "Patient-GAT: Sarcopenia Prediction using Multi-modal Data Fusion and Weighted Graph Attention Networks," in *Proceedings of the 38th ACM/SIGAPP Symposium on Applied Computing*, 2023, pp. 614-617.

[25] D. Ouyang *et al.*, "Integration of multi-omics data using adaptive graph learning and attention mechanism for patient classification and biomarker identification," *Computers in Biology and Medicine,* vol. 164, p. 107303, 2023.

[26] A. Ma *et al.*, "Single-cell biological network inference using a heterogeneous graph transformer," *Nature Communications,* vol. 14, no. 1, p. 964, 2023.

[27] B. Lei *et al.*, "Alzheimer's disease diagnosis from multi-modal data via feature inductive learning and dual multilevel graph neural network," *Medical Image Analysis,* vol. 97, p. 103213, 2024.

[28] H. Zhou, L. He, B. Y. Chen, L. Shen, and Y. Zhang, "Multi-modal diagnosis of Alzheimer's disease using interpretable graph convolutional networks," *IEEE Transactions on Medical Imaging,* 2024.

[29] Z. Zhang *et al.*, "Pathology-genomic fusion via biologically informed cross-modality graph learning for survival analysis," *arXiv preprint arXiv:2404.08023,* 2024.

[30] B. Patel *et al.*, "Explainable multimodal graph isomorphism network for interpreting sex differences in adolescent neurodevelopment," *Applied Sciences,* vol. 14, no. 10, p. 4144, 2024.

[31] G. Qu, Z. Zhou, V. D. Calhoun, A. Zhang, and Y.-P. Wang, "Integrated brain connectivity analysis with fMRI, DTI, and sMRI powered by interpretable graph neural networks," *Medical Image Analysis,* vol. 103, p. 103570, 2025.

[32] M. Sundararajan, A. Taly, and Q. Yan, "Axiomatic attribution for deep networks," in *International conference on machine learning*, 2017: PMLR, pp. 3319-3328.

[33] S. M. Lundberg and S.-I. Lee, "A unified approach to interpreting model predictions," *Advances in neural information processing systems,* vol. 30, 2017.
[34] D. G. Garson, "Interpreting neural network connection weights," 1991.
[35] Z. Ying, D. Bourgeois, J. You, M. Zitnik, and J. Leskovec, "Gnnexplainer: Generating explanations for graph neural networks," *Advances in neural information processing systems,* vol. 32, 2019.
[36] C. Cangea, P. Veličković, N. Jovanović, T. Kipf, and P. Liò, "Towards sparse hierarchical graph classifiers," *arXiv preprint arXiv:1811.01287,* 2018.
